# Single‐Molecule Fluorescence Detection of a Synthetic Heparan Sulfate Disaccharide

**DOI:** 10.1002/cphc.201600750

**Published:** 2016-09-14

**Authors:** Charlotte E. Dalton, Steven D. Quinn, Aidan Rafferty, Michael J. Morten, John M. Gardiner, Steven W. Magennis

**Affiliations:** ^1^ The School of Chemistry and Manchester Institute of BiotechnologyThe University of Manchester131 Princess StreetManchesterM1 7DNUK; ^2^ WestCHEM, School of ChemistryUniversity of GlasgowJoseph Black Building, University AvenueGlasgowG12 8QQUK

**Keywords:** carbohydrate, fluorescence, heparan sulfate, single-molecule studies, vesicle

## Abstract

The first single‐molecule fluorescence detection of a structurally‐defined synthetic carbohydrate is reported: a heparan sulfate (HS) disaccharide fragment labeled with Alexa488. Single molecules have been measured whilst freely diffusing in solution and controlled encapsulation in surface‐tethered lipid vesicles has allowed extended observations of carbohydrate molecules down to the single‐molecule level. The diverse and dynamic nature of HS–protein interactions means that new tools to investigate pure HS fragments at the molecular level would significantly enhance our understanding of HS. This work is a proof‐of‐principle demonstration of the feasibility of single‐molecule studies of synthetic carbohydrates which offers a new approach to the study of pure glycosaminoglycan (GAG) fragments.

## 1 Introduction

Single‐molecule fluorescence spectroscopy has revolutionized the study of biological molecules.^1^ In contrast to nucleic acids and proteins, for which single‐molecule detection has been well exploited, applications to structurally‐defined carbohydrates have not yet been reported, in part owing to the requirement for non‐trivial synthetic chemistry. While carbohydrates have been modified with fluorescent dyes for ensemble studies,^2^, ^3^ this does not guarantee the homogeneity and photostability required for single‐molecule detection. To date, there have been only a few reports of the single‐molecule detection of carbohydrates. The super‐resolution imaging of cells containing metabolically‐labeled glycans was reported,^4^, ^5^ whereby any modified glycan on the cell surface can be covalently labeled with a fluorophore using click chemistry.^6^, ^7^ In addition, the conformational change induced in a protein by the simple sugar maltose was also reported, though the photophysics of the dye–maltose conjugate were not characterized.^8^ In contrast, we are interested in the interactions of complex, structure‐specific carbohydrate ligands with biomedically‐important protein targets using well‐defined fluorescent glycoconjugates.

In this work, we demonstrate the fluorescent labeling, full photophysical characterization and single‐molecule detection of a chemically synthesized, structurally‐specific heparan sulfate (HS) disaccharide. The glycosaminoglycan (GAG) polysaccharide HS, along with the related GAG heparin, is known to bind a wide variety of proteins including chemokines and growth factors. However, owing to the structural complexity of HS, structural requirements for protein binding with regards to variable sulfation patterns and sequence lengths are ill defined. Although digest products of native HS can be used to investigate HS properties, these are necessarily heterogeneous. Chemical synthesis of HS oligosaccharides allows full control of the structure of resulting oligosaccharides, for example, enabling variable defined lengths^9^, ^10^, ^11^ and programmable sulfation.^12^ NMR has been employed in recent years for structural investigation of HS fragment‐protein interactions, to probe HS oligosaccharide protein‐bound conformation,^13^ or identification of binding site amino acids.^14^ Similarly, X‐ray crystallography has been utilized to study HS–protein complexes and also more complex systems such as the FGF1–heparin–FGF receptor.^15^ Whilst these ensemble techniques have greatly advanced the understanding of HS–protein interactions, single‐molecule methods provide unique information because such measurements avoid ensemble averaging. This is of particular relevance to dynamic and/or heterogeneous processes. A better understanding of the structural requirements for HS–protein binding could facilitate drug design for numerous conditions in which HS–protein interactions are implicated,^16^ for example cancers^17^ and viral infections.^18^

## 2 Results and Discussion

Owing to the similarity between the protein‐binding regions (NS domains) of HS and the related GAG heparin, the major glucosamine‐iduronic acid heparin disaccharide is a useful model of HS and was thus chosen for fluorescent labeling. This fragment is a key component in biologically‐active sequences. Amine tags have been used for the functionalization of synthetic HS oligosaccharides^19^, ^20^, ^21^, ^22^ (e.g. for microarray attachment, solid phase support).

**Scheme 1:**
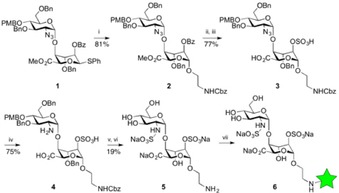
Synthesis of labeled disaccharide **6**. *Reagents*: i) *N*‐*Z*‐ethanolamine, NIS, AgOTf, CH_2_Cl_2_, 4 Å mol. sieves; ii) LiOH, THF/MeOH/H_2_O; iii) SO_3_.Py, pyridine, RT; iv) PMe_3_ in THF, NaOH, THF, RT; v) SO_3_.Py, Et_3_N, pyridine, RT; vi) H_2_, Pd(OH)_2_, EtOH/H_2_O; vii) Alexa Fluor 488 SDP ester (green, see SI for full structure), H_2_O/DMSO, pH 8.

We introduced a short protected amine‐terminated reducing end tag from disaccharide **1,** which we previously deployed as the core iterative unit enabling synthesis of synthetic HS fragments^23^ (Scheme 1). Glycosylation of **1** provided **2** with high anomeric selectivity and **2** was then elaborated into sulfated disaccharide **5** with a free amino terminus. Synthesis of such pure sulfated GAG fragments is non‐trivial and access to a substrate suitable for fluorescent labelling required development. Reaction of **5** with Alexa Fluor 488 SDP ester at pH 8 afforded the desired fluorescent conjugate **6** (n.b. 0.6 equivalents of dye were used to avoid the need for separation of disaccharide and free dye).

**Figure 1:**
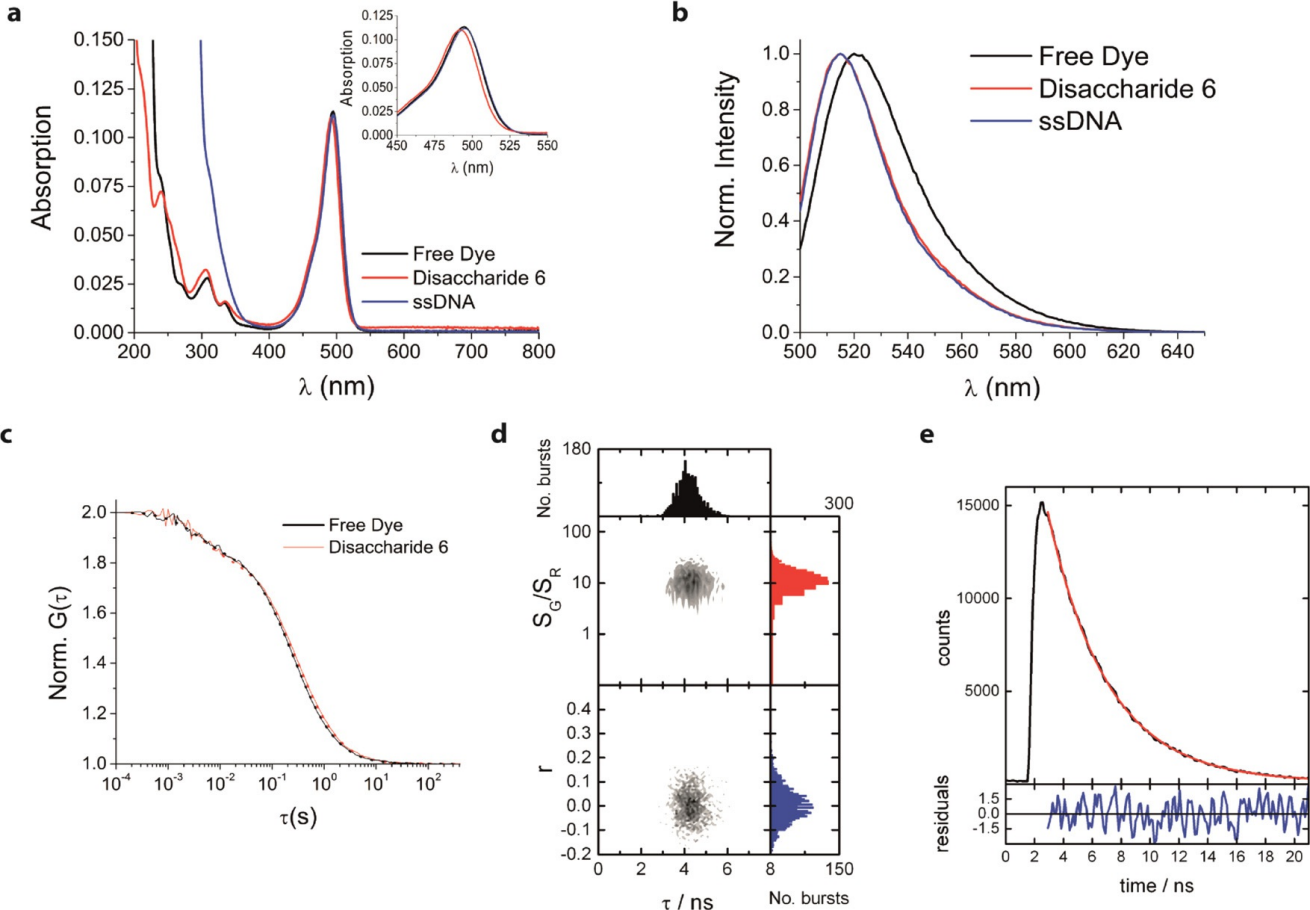
Ensemble and single‐molecule detection of freely‐diffusing disaccharides. a) Absorption spectra and b) Normalized fluorescence emission spectra of free Alexa488 SDP, Alexa488‐labelled disaccharide **6** and Alexa488‐labelled ssDNA. The inset of (a) shows a smaller region of the same spectra for free dye and **6**. c) Fluorescence correlation spectra of **6** and free dye. Correlation curves (line) and fits (dots) are shown. See the Supporting Information for fitting parameters. d) Multi‐parameter confocal microscopy of single molecules of **6**. 2D histogram of fluorescence lifetime (*τ*) versus ratio of signal in green detection channel (*S*_G_) to that in the red channel (*S*_R_), and the fluorescence anisotropy (*r*). e) Sub‐ensemble analysis of single molecule bursts within the region *τ*=3.0–5.6 ns and *S*_G_/*S*_R_=4.8–42.7 in (d). The decay (black line), tail fit to a single‐exponential decay (red line) and the residuals (in blue) are shown, with lifetime of 4.1±0.1 ns; Samples were prepared in 20 mm Tris, 10 mm MgCl_2_, pH 7.5 buffer.

The structure of **6** was confirmed by mass spectrometry (Figure S1) and proton NMR, which also indicated no remaining free dye (Figure S2). Access to small‐scale fluorescent GAG fragment conjugates is challenging and we believe that NMR characterization of such conjugates has not been previously reported. This is significant here as the observed NMR coupling constants imply that the iduronic acid (IdoA) residue exists in the same conformation (^1^C_4_) in both the labeled and unlabeled species. The conformation flexibility of l‐idoA is significant in the binding of GAG fragments and establishing by NMR that a terminal l‐idoA is not affected by label conjugation is valuable. Prior to single‐molecule evaluation, **6** was studied using ensemble steady‐state optical spectroscopy, along with the free dye and Alexa488‐labelled single‐stranded DNA molecules (ssDNA). The absorption spectrum of **6** is very similar to that of the free dye, with only minor changes in peak positions and profile (Figure 1 a). In contrast, the emission spectrum of **6** exhibits a blue shift in comparison to the free dye (Figure 1 b). Importantly, the absorption and emission spectrum of **6** are essentially identical to that of Alexa488 attached to DNA, indicating a similar perturbation of Alexa488 upon attachment to the disaccharide.

Single‐molecule measurements were first made on the disaccharide freely diffusing in aqueous solution by using confocal microscopy. Fluorescence correlation spectroscopy (FCS) of **6** produced a correlation curve that could be fitted using the same model^24^ as for the free dye, incorporating fluctuations caused by diffusion and triplet formation (Figure 1 c). The recovered diffusion time for **6** is 307±4 μs, whereas the value that we measured for the free Alexa488 dye is 254±3 μs and that for the related rhodamine 110 dye is 200±2 μs under identical conditions. This corresponds to a 27 % and 53 % increase in hydrodynamic radii for Alexa488 and **6**, respectively, in comparison to rhodamine 110. The similarity in photon count rate per molecule for **6** (44±3 kHz molecule^−1^) and the free dye (40±5 kHz molecule^−1^) agrees with the small differences observed in the ensemble data.

We next performed multi‐parameter fluorescence detection (MFD) to look at individual labeled disaccharides. The MFD method uses a confocal microscope to study freely‐diffusing molecules in multiple detection channels.^25^ The majority of the Alexa488 emission was collected in two of the detection channels (green), while the tail of the emission spectrum overlapped with the red detection channels. Figure 1 d shows a plot of the fluorescence lifetime (*τ*) of molecules detected in the green channels versus the ratio of the signal in the green detection channels (*S*_G_) to that in the red detection channels (*S*_R_), and the fluorescence anisotropy (*r*) in the green channel. Figure 1 d reveals that only one population of fluorescent molecules is present, with the lifetime centered on 4.1 ns and anisotropy close to 0. The lifetime of 4.1 ns indicates that the Alexa488 dye is unquenched, while the low anisotropy indicates that it is able to rotate freely.^26^ The data are very similar to those collected for DNA labeled with Alexa488, which has been well‐characterized previously.^27^ A sub‐ensemble analysis of this population (Figure 1 e) shows that the fluorescence decay can be fitted to a single‐exponential decay with a lifetime of 4.1 ns, indicating a single dye environment and the absence of dynamics on the millisecond timescale. The presence of just one population indicates that there is a single labeled species (in agreement with NMR and mass spectrometry analysis of **6**). It is possible, however, that alternative conformations of the disaccharide are present (e.g. via ring flipping), but that these do not lead to changes in the properties of the fluorescent label.

**Figure 2:**
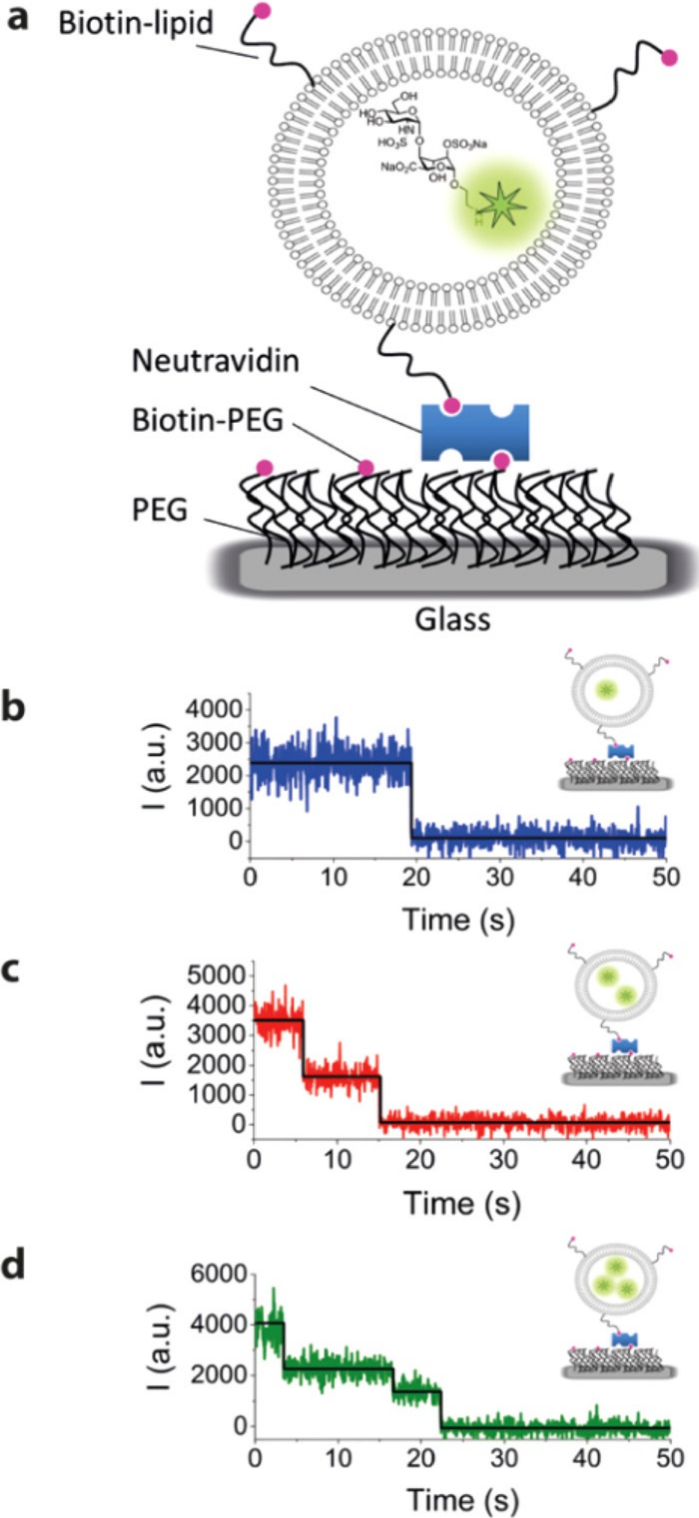
Single‐molecule TIRF detection of immobilized disaccharides. a) Immobilization scheme for **6** in vesicles. Representative 1‐, 2‐ and 3‐step photobleaching traces (b, c and d, respectively); the fits are shown as solid black lines. Experiments were performed in 20 mm Tris, 6 % (w/v) glucose, 1 mg mL^−1^ glucose oxidase, 0.04 mg mL^−1^ catalase, 2 mm trolox, pH 8 buffer. Vesicles were prepared in the same buffer.

To study the behavior of the individual disaccharides over longer timescales, we encapsulated them into nanoscale vesicles, which were then immobilized onto a glass coverslide through biotin‐neutravidin interactions. This approach has been widely used for studying single molecules, avoiding the need for covalent attachment of the molecule of interest directly to a surface.^28^ It allows the partitioning of molecules between the aqueous vesicle interior and exterior, and the encapsulated molecules can often diffuse freely within the nanocontainer. We made small unilamellar vesicles (SUVs) composed of 98 mol % egg‐PC and 2 mol % biotinyl‐PE by extrusion. Dynamic light scattering of vesicles formed in the presence of disaccharide **6** showed them to have an average diameter of 120 nm, with a narrow distribution of sizes (Figure S3). A schematic of the immobilization scheme used for attaching vesicles to a glass substrate is shown in Figure 2 a. The biotinylated lipids allow each vesicle to bind a single neutravidin protein, which is in turn bound to a single biotinylated‐PEG chain adsorbed onto the glass surface. The surface was imaged using wide‐field total internal reflection fluorescence (TIRF) microscopy, allowing long‐time observation of hundreds of single molecules in parallel. Vesicles were prepared in solution containing 500 nm disaccharide. To ensure fluorescence signals originated from encapsulated disaccharide, varying concentrations of vesicles were added to the slide, resulting in a corresponding change in the number of fluorescent spots (Figure S4).

The number of molecules inside a particular vesicle can be measured by recording the number of bleaching steps in time traces of the fluorescence signal from individual spots. This revealed levels of constant intensity, followed by stepwise photobleaching of the fluorophores (Figures 2 b–d). The stability of the signals before a bleaching event (i.e. the absence of blinking) allows a reliable estimate of the numbers of encapsulated molecules. Traces predominantly displayed one‐step bleaching events (Figure 2 b) with fewer two‐ and three‐step bleaching events observed (Figures 2 c and 2 d, respectively). Thus most detected vesicles prepared using a disaccharide concentration of 500 nm contained only one disaccharide. There were no instances of bleaching events beyond four steps. Preparation of vesicles containing 1 μm disaccharide gave predominantly two‐step bleaching events; traces showing up to four‐ or five‐step bleaching were also recorded (Figure S5). Increasing the amount of encapsulated disaccharide further (10 μm) produced exponential‐like photobleaching decays at early time, suggesting the presence of large numbers of dye molecules (>10) within the vesicles, followed by stepwise bleaching at longer times (Figure S6). An evaluation of the fluorescence on‐times as a function of irradiance showed that at 260 W cm^−2^ the dyes were photobleaching over timescales typically <10 s, while at 65 W cm^−2^ and 130 W cm^−2^ the dyes were longer lived (Figure S7).

The stability of the time traces is notable, with no dynamics on the time scales studied. This is further evidence that the Alexa488 dye is not perturbed by attachment to the sugar, since interactions with the sugar might lead to dark states in which the dye emission is quenched. Furthermore, we believe that direct lipid–carbohydrate interactions must be absent or very weak, since addition of **6** to immobilized vesicles in solution resulted in no binding to either vesicles or the PEG surface. Presumably, this also means that the disaccharides are free inside the vesicles.

## 3 Conclusions

We have reported the synthesis and detection of fluorescently‐labeled HS disaccharide conjugate **6**, freely diffusing and immobilized, at the single‐molecule level. The favorable fluorescent properties of the Alexa488 label were maintained upon attachment to the sugar, indicating that this approach should be suitable for other labeled GAG oligosaccharides of varying length, allowing a wide range of biological systems to be probed.

Encapsulating labeled disaccharides in lipid vesicles, as shown via stepwise photobleaching, allows long‐time observation. The lack of direct lipid‐sugar interaction encourages the possibility of using such vesicles as artificial cells, by incorporating additional functionality to the lipid bilayer as models for GAG–protein interactions.

This work forms the basis of a new method for the investigation of the behavior of structure‐specific synthetic HS‐fragments at the single molecule level, which could provide information that is currently hidden from ensemble methods such as NMR and X‐ray crystallography. It is a proof‐of‐principle demonstration of the single‐molecule fluorescence detection of synthetic carbohydrates, which should lead to new approaches for analyzing the molecular interactions of this important class of biomolecule at the single‐molecule level free in solution and when encapsulated.

## Experimental Section

Full experimental details are provided in the Supporting Information.

## Supporting information

As a service to our authors and readers, this journal provides supporting information supplied by the authors. Such materials are peer reviewed and may be re‐organized for online delivery, but are not copy‐edited or typeset. Technical support issues arising from supporting information (other than missing files) should be addressed to the authors.

Supplementary
