# Supplementary material for: Single‐Molecule Fluorescence Detection of a Synthetic Heparan Sulfate Disaccharide
Source: Chemphyschem. 2016 Sep 14;17(21):3442–6. doi: 10.1002/cphc.201600750 (PMC5111599; doi:10.1002/cphc.201600750)
Supplement: Supplementary file 1 — Supplementary [file CPHC-17-3442-s001.pdf]

CHEMPHYSCHEM

Supporting Information

Single-Molecule Fluorescence Detection of a Synthetic Heparan Sulfate Disaccharide

Charlotte E. Dalton+,[a] Steven D. Quinn+,[b] Aidan Rafferty,[b] Michael J. Morten,[b] John M. Gardiner,*[a] and Steven W. Magennis*[b]

cphc_201600750_sm_miscellaneous_information.pdf

Supporting Information for

Single-molecule fluorescence detection of a synthetic heparan sulfate disaccharide

Charlotte E. Dalton,[1,†] Steven D. Quinn,[2,†] Aidan Rafferty,[2] Michael J. Morten,[2] John M. Gardiner[1]*, Steven W. Magennis[2]*

*Correspondence to Dr. John Gardiner or Dr. Steven Magennis

This PDF includes:

Synthesis and Characterization of compounds **2-6**
Supplementary Methods
Supplementary Figs. 1-7
NMR spectra: spectrum 1 – spectrum 12
References

Synthesis and characterization

General synthetic experimental detail

IR spectra were recorded for solid samples using a Bruker Alpha-P ATR spectrometer. NMR spectra were recorded for solutions in $CDCl_3$, CD_3COOD, or D_2O on a Bruker Avance instrument (400 MHz) and were referenced to the residual solvent signal. Assignments were determined using COSY and HMQC experiments. Where resonances in the ^{13}C NMR spectrum are not assignable due to spectral overlap, the multiplicity of the carbon is given (from DEPT135). Coupling constants (J values) are quoted to the nearest 0.1 Hz, and are given as measured *i.e.* not corrected to be alike for coupled protons. Protons on the iduronic acid residue are denoted H-1, H-2 etc, protons on the glucosamine residue H-1', H-2' etc. Accurate mass measurements were obtained using a Thermofisher LTQ Orbitrap XL instrument with nanospray ionisation (EPSRC National Mass Spectrometry Facility, Swansea). Optical rotations were measured using a Bellingham and Stanley ADP410 or an Optical Activity AA-1000 polarimeter, and are given in units of deg cm^3 g^{-1} dm^{-1} with c quoted in g/100 mL. Analytical thin layer chromatography (TLC) was performed on Merck TLC Silica gel 60 plates (aluminium backed, UV_{254} fluorescent indicator) with visualisation using UV light and/or cerium molybdate stain. Preparative column chromatography was performed using Sigma-Aldrich silica gel (technical grade, 60 Å, 220–240 mesh, 35–75 µm). All chemicals and solvents used were of standard laboratory grade unless otherwise specified. Dichloromethane was dried using calcium hydride under nitrogen. Other anhydrous solvents were used as purchased. Compositions of solvent mixtures are quoted as ratios of volume. Molecular sieves were activated by drying under high vacuum for 5 h, followed by storage in an oven at 200 °C. Organic solutions were dried with anhydrous magnesium sulfate and concentrated by rotary evaporation under reduced pressure.

Methyl [2-(carboxybenzyl-amino)-ethanyl-4-*O*-(2-azido-3,6-di-*O*-benzyl-4-*p*-methoxybenzyl-2-deoxy-α-D-glucopyranosyl)-2-*O*-benzoyl-3-*O*-benzyl-α-L-idopyranoside] uronate **2**

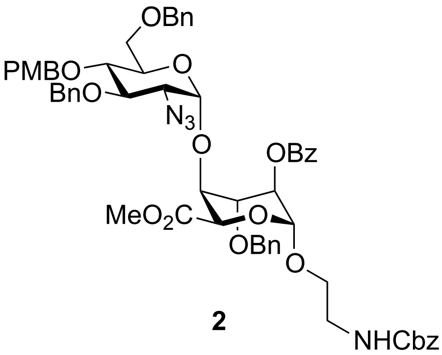

2

Disaccharide **1** (386 mg, 0.39 mmol) and N-*Z*-aminoethanol (92 mg, 0.47 mmol) were dissolved in toluene, concentrated *in vacuo*, and the process repeated twice. The mixture was dried under high vacuum for 1 h, then dissolved in anhydrous CH_2Cl_2 (5 mL) under N_2 and cooled to 0 °C. Molecular sieves (~300 mg, 4 Å, powdered, activated) were added and the reaction was stirred at 0 °C for 30 min. NIS (193 mg, 0.86 mmol) was added and the resulting pale red solution stirred at 0 °C for 30 min. AgOTf (10 mg, 40 μmol) was added and the reaction stirred at 0 °C for 30 min, then at RT for 1 h. TLC (2:1 hexane/EtOAc) showed no remaining disaccharide starting material. $Na_2S_2O_3$ (0.5 g) and $NaHCO_3$ (0.5 g) in water (10 mL) were added and the reaction stirred for 10 min at RT. The reaction mixture was filtered through Celite, the phases separated and the aqueous phase washed with CH_2Cl_2 (2 x 20 mL). The combined organic phases were dried and concentrated *in vacuo* to an orange oil. Column chromatography (2:1 hexane/EtOAc) afforded the *title compound* **2** as a yellow foam (340 mg, 0.32 mmol, 81%); R_f 0.24 (2:1 hexane/EtOAc); IR (ν_{max}/cm^{-1}): 2920 (C–H), 2106 (N_3), 1717 (C=O); MS (ES^+) found *m/z* 1084 $[M+NH_4]^+$, HRMS found 1084.4558, $C_{59}H_{66}N_5O_{15}$ $[M+NH_4]^+$ requires 1084.4550; $[\alpha]_D$ = –22.5 (c = 0.45, CH_2Cl_2); δ_H (400 MHz, $CDCl_3$): 8.05–8.03 (2 H, m, Ar*H*), 7.36–7.15 (21 H, m, Ar*H*), 7.15–7.07 (2 H, m, Ar*H*), 7.03–6.99 (2 H, m, Ar*H*), 6.82–6.77 (2 H, m, Ar*H*), 5.18 (1 H, t, *J* = 5.3 Hz, N*H*), 5.15 (1 H, br s, H-1), 5.09 (1 H, t, *J* = 2.1 Hz, H-3), 5.02 (2 H, s, 2 x C*H$_2$*Ar), 4.84 (1 H, d, *J* = 11.6 Hz, C*H$_2$*Ar), 4.80-4.75 (2 H, m, H-1’, H-5), 4.70 (1 H, d, *J* = 11.6 Hz, C*H$_2$*Ar) 4.58–4.54 (2 H, m, 2 x C*H$_2$*Ar), 4.43 (1 H, d, *J* = 11.3 Hz, C*H$_2$*Ar), 4.39 (1 H, d, *J* = 10.5 Hz, C*H$_2$*Ar) 4.18 (1 H, d, *J* = 10.8 Hz, C*H$_2$*Ar), 4.12 (1 H, t, *J* = 2.4 Hz, H-2), 4.07 (1 H, t, *J* = 3.2 Hz, H-4), 3.95 (1 H, d, *J* = 10.8 Hz, C*H$_2$*Ar), 3.88–3.85 (1 H, m, H-5’), 3.82–3.77 (2 H, m, H-6’, C*H$_2$* handle), 3.75 (3 H, s, OC*H$_3$*), 3.68–3.58 (6 H, m, H-6’’, H-4, C*H$_2$* handle, C(O)OC*H$_3$*), 3.49 (1 H, t, *J* = 9.6 Hz, H-3’), 3.39–3.35 (2 H, m, C*H$_2$* handle), 3.18 (1 H, dd, *J* = 10.2, 3.4 Hz, H-2’); δ_C (100 MHz, $CDCl_3$): 169.3 (C=O), 165.6 (C=O), 159.3 (C=O), 156.4 (Ar C), 138.0 (Ar C), 137.9 (Ar C), 137.5 (Ar C), 136.6 (Ar C), 133.3 (Ar CH), 130.5 (Ar CH),

130.0 (Ar CH), 129.5 (Ar CH), 128.8 (Ar CH), 128.6 (Ar CH), 128.5 (Ar CH), 128.4 (2 x Ar CH), 128.1 (2 x Ar CH), 128.0 (2 x Ar CH), 127.8 (Ar CH), 127.7 (2 x Ar CH), 113.7 (Ar CH), 99.9 (C-1'), 99.2 (C-1), 80.0 (C-3'), 77.5 (CH), 75.6 (C-4), 74.7 (CH_2), 74.5 (CH_2), 73.6 (CH_2), 73.5 (C-2), 72.6 (CH_2), 71.8 (C-5'), 68.0 (C-3), 67.8 (CH_2), 67.7 (C-5), 66.7 (CH_2), 63.7 (C-2'), 55.3 (CH_3), 52.4 (CH_3), 40.8 (CH_2).

2-(Carboxybenzyl-amino)-ethanyl-[4-*O*-(2-azido-3,6-di-*O*-benzyl-4-*O*-*p*-methoxybenzyl-2-deoxy-α-D-glucopyranosyl)-2-sulfo-3-*O*-benzyl-α-L-idopyranoside] uronic acid **3**

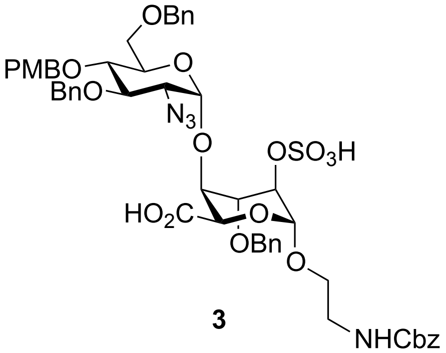

3

To a stirred solution of disaccharide **2** (334 mg, 0.31 mmol) in THF (4 mL) was added MeOH (1 mL) and the reaction was cooled to 0 °C. LiOH monohydrate (52 mg, 1.25 mmol) was dissolved in water (2 mL) and added dropwise. The reaction mixture was allowed to gradually warm to RT and stirred for 2 h. TLC (95:5 CH_2Cl_2/MeOH) showed no remaining disaccharide starting material. HCl (300 μL, 1 M) was added and the solvents removed *in vacuo* to give a white solid. This crude product was dissolved in anhydrous pyridine (6 mL), sulfur trioxide-pyridine complex was added (277 mg, 1.75 mmol) and the reaction stirred at RT for 20 h. TLC (92.5:7.5 CH_2Cl_2/MeOH) showed no remaining disaccharide starting material. The solvent was removed *in vacuo* to give a white solid. Column chromatography (92.5:7.5 CH_2Cl_2/MeOH) afforded the *title compound* **3** as a yellow foam (242 mg, 0.24 mmol, 77% over two steps). Sulfated product **3** was determined to be approx. 95% pure by NMR, but broadening of the peaks prevented assignment, and the material was used as is in the next step. Data collected for **3**: R_f 0.18 (9:1 CH_2Cl_2/MeOH); MS (ES^-) found *m/z* 513 $[M–2H]^{2-}$, HRMS found 513.1595, $C_{51}H_{54}N_4O_{17}S$ $[M–2H]^{2-}$ requires 513.1608.

2-(Carboxybenzyl-amino)-ethanyl-[4-*O*-(2-amino-3,6-di-*O*-benzyl-4-*O*-*p*-methoxybenzyl-2-deoxy-α-D-glucopyranosyl)-2-sulfo-3-*O*-benzyl-α-L-idopyranoside] uronic acid **4**

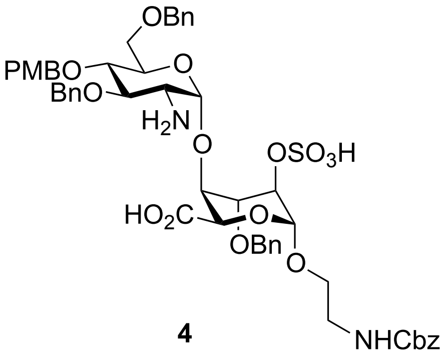

4

To a stirred solution of disaccharide **3** (240 mg, 0.24 mmol) in THF (5 mL) was added aqueous NaOH (2 mL, 0.1 M). PMe_3 in THF (1.0 mL, 1.0 M) was added dropwise. The reaction mixture was stirred for 2 h at RT. TLC (9:1 CH_2Cl_2/MeOH) showed no remaining disaccharide starting material. Aqueous HCl (0.1 M) was added until the solution reached neutral pH, and the solvents were removed *in vacuo* to give a white glass. Size exclusion chromatography on Sephadex® LH-20 resin (1:1 CH_2Cl_2/MeOH) afforded the *title compound* **4** as a clear glass (180 mg, 0.18 mmol, 75%); R_f 0.44 (9:1 CH_2Cl_2/MeOH); IR (ν_{max}/cm^{-1}): 3367 (O–H), 2920 (C–H), 1715 (C=O); MS (ES$^-$) found *m/z* 1001 [M–H]$^-$, HRMS found 1001.3387, $C_{51}H_{57}N_2O_{17}S$ [M–H]$^-$ requires 1003.3372; $[\alpha]_D$ = +16.2 (c = 0.98, CH_2Cl_2); δ_H (400 MHz, CD_3CO_2D): 7.31–7.13 (21 H, m, Ar*H*), 6.95–6.89 (2 H, m, Ar*H*), 6.73–6.66 (2 H, m, Ar*H*), 5.28 (1 H, d, *J* = 2.4 Hz, H-1'), 5.24 (1 H, s, H-1), 5.08–5.01 (2 H, m, 2 x CH_2Ar), 4.94–4.89 (2 H, m, H-3, CH_2Ar), 4.77–4.70 (3 H, m, H-5, 2 x CH_2Ar), 4.62–4.42 (5 H, m, 5 x CH_2Ar), 4.26 (1 H, s, H-2), 4.12 (1 H, s, H-4), 3.93 (1 H, t, *J* = 9.6 Hz, H-3'), 3.82–3.70 (4 H, m, H'-4, H'-5, H'-6, CH_2 handle), 3.65–3.54 (5 H, m, OCH_3, CH_2 handle, H-6'), 3.47 (1 H, d, *J* = 10.0 Hz, H-2'), 3.39–3.29 (2 H, m, 2 x CH_2 handle); δ_C (100 MHz, CD_3CO_2D): 159.4 (C=O), 157.7 (C=O), 138.1 (Ar C), 137.6 (Ar C), 137.6 (Ar C), 136.5 (Ar C), 129.8 (Ar CH), 129.7 (Ar CH), 128.4 (Ar CH), 128.3 (Ar CH), 128.3 (Ar CH), 128.2 (Ar CH), 128.0 (Ar CH), 127.9 (Ar CH), 127.8 (Ar CH), 127.7 (Ar CH), 127.5 (Ar CH), 127.3 (Ar CH), 113.6 (Ar CH), 98.6 (C-1), 91.5 (C-1'), 77.8 (CH), 77.2 (C-3'), 74.8 (CH_2), 74.3 (CH_2), 73.2 (CH_2), 71.7 (CH), 71.4 (CH_2), 69.7 (C-2), 69.5 (C-4), 67.4 (CH_2), 67.1 (CH_2), 66.9 (CH_2), 54.5 (CH_3), 54.0 (C-2'), 40.5 (CH_2).

2-amino-ethanyl-[4-*O*-(2-amino-2-deoxy-α-D-glucopyranosyl)-2-sulfo-α-L-idopyranoside] uronic acid trisodium salt **5**

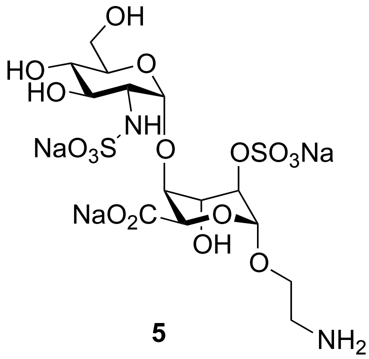

5

To a stirred solution of disaccharide **4** (189 mg, 0.19 mmol) in dry pyridine (3 mL) was added sulfur trioxide-pyridine complex (300 mg, 1.89 mmol) and Et_3N (500 µL) and the reaction was stirred for 2 h at RT. TLC (9:1 CH_2Cl_2/MeOH) showed no remaining disaccharide starting material. Saturated aq. $NaHCO_3$ was added dropwise until effervescence ceased. The solvent was removed *in vacuo* to give an oil, size exclusion chromatography on Sephadex LH-20 resin (CH_2Cl_2) afforded the *N*-sulfated disaccharide as a clear glass (83 mg, 76.4 µmol). Data collected for this intermediate: R_f 0.23 (9:1 CH_2Cl_2/MeOH); MS (ES^-) found *m/z* 540 $[M–2H]^{2-}$, HRMS found 540.1434, $C_{51}H_{56}N_2O_{20}S_2$ $[M–2H]^{2-}$ requires 540.1439.

The intermediate N-sulfated disaccharide (37 mg, 34 µmol) was then dissolved in EtOH/H_2O (1:1, 2 mL), Pd/C (45 mg) was added under N_2, the reaction purged with H_2/vacuum and the solution stirred under an atmosphere of H_2 for 2 d. The solution was filtered through Celite with H_2O/EtOH and the solvent removed *in vacuo* to give a white glass (32 mg) which was purified on Dowex 50WX4 Na^+ resin (eluent H_2O) to give the *title compound* **5** as a white powder after lyophilization (10 mg, 16 µmol, 19% over two steps); R_f 0.09 (6:5:3:1 ethyl acetate/pyridine/H_2O/acetic acid); IR (ν_{max}/cm^{-1}): 3360 (O–H), 1763 (C=O); HRMS found 557.0595, $C_{14}H_{25}N_2O_{17}S_2$ $[M\text{-}3Na+2H]^-$ requires 557.0600; $[\alpha]_D$ = +71.7 (c = 0.1, CH_2Cl_2); δ_H (400 MHz, D_2O): 5.41 (1 H, t, *J* = 2.8 Hz, H-1’), 5.12 (1 H, t, *J* = 3.0 Hz, H-1), 4.54 (1 H, t, *J* = 3.1 Hz, H-5), 4.30–4.27 (1 H, m, H-2), 4.22–4.19 (1 H, m, H-3), 4.09 (1 H, t, *J* = 3.2 Hz, H-4), 4.01 (1 H, ddd, *J* = 11.5, 5.7, 3.6 Hz, H-6’), 3.83–3.75 (4 H, m, H-5’, H-6’, 2 x CH_2 handle), 3.61 (1 H, t, *J* = 9.8 Hz, H-3’), 3.44 (1 H, t, *J* = 9.3 Hz, H-4’), 3.30–3.23 (1 H, m, 2 x CH_2 handle), 3.20 (1 H, dd, *J* = 10.4, 3.4 Hz, H-2’); δ_C (100 MHz, D_2O): 99.3 (C-1), 96.5 (C-1’), 76.5 (C-2), 75.7 (C-4), 71.7 (C-5’), 71.1 (C-3’), 69.9 (C-4’), 69.3 (C-5’ and C-3), 64.4 (C-6), 60.3 (CH_2), 58.0 (C-2’), 39.2 (CH_2).

Synthesis of labeled disaccharide **6**

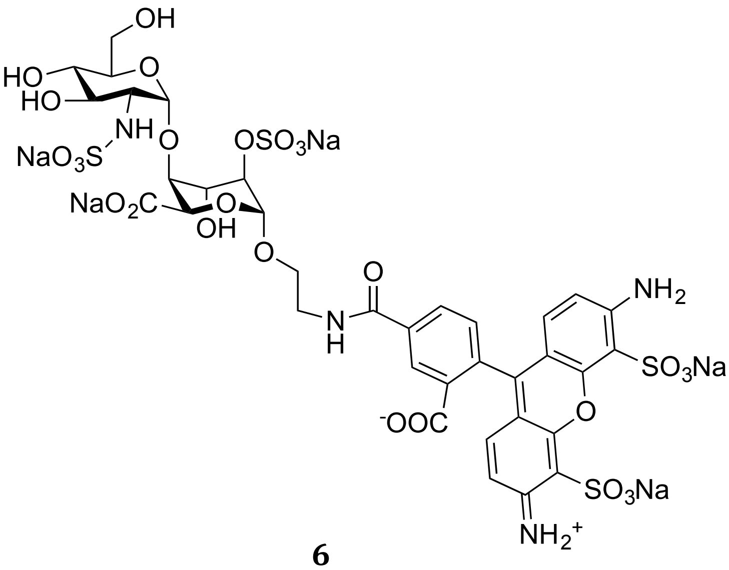

6

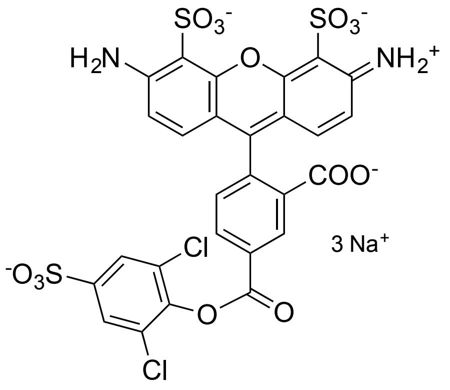

AF488 5-SDP ester (free dye)

To disaccharide **5** (1.38 mg, 2.2 μmol) in 0.1 M $NaHCO_3$ (100 μL, pH 8) was added Alexa Fluor 488 5-SDP ester (Life Technologies, 1.0 mg, 1.32 μmol, 0.6 equivalents, in 50 μL DMSO*). The resulting mixture was shaken for 96 h protected from light (aluminum foil). The resulting solution was lyophilized to give labeled disaccharide **6** (and residual unlabeled **5**) as an orange solid. Proton NMR and HRMS data were collected, see Figures S2 and S3.

* *N.B.* 0.6 equivalents of dye were used to avoid potential problematic separation of free dye and disaccharides, which have similar molecular weights. Single use (ampule) deuterated DMSO (Cambridge Isotopes) was used to ensure purity of the solvent.

Supplementary Methods

Steady-state ensemble spectroscopy

Absorption and emission spectra were acquired on Cary 60 (Agilent Technologies) and Fluoromax (HORIBA Scientific) spectrometers, respectively. Emission spectra were recorded under magic angle conditions and background fluorescence from the solvent was negligible.

The sequence of the labeled ssDNA discussed in the manuscript (see Fig. 1) is 5' TGG CGA CGG CAG CGA GGC TTA GCG GCA AAA AAA AAA AAA AAA AAA AAA AAA AAA

AAA AGC CGC X, where X = T-(Alexa 488) at position 64. The oligo was supplied by Purimex GmbH (Germany).

Fluorescence Correlation Spectroscopy

FCS measurements were performed on the same confocal microscope used for MFD measurements (see below). Samples were prepared in Nunc Lab-Tek II chambered cover glasses (4 wells, VWR, UK) and excited via the linearly polarized light of a pulsed 488 nm laser (Becker & Hickl). The laser light was focused into the sample solution by a water immersion objective lens (UPLAPO 60x, NA = 1.2, Olympus, UK) mounted in an inverted microscope (IX-71, Olympus, UK). The average laser power of the focused beam at the sample was ca. 170 μW. As each molecule traverses the detection volume, a burst of fluorescence photons are generated. The emission photons were subsequently divided into parallel and perpendicular components via a polarizing beamsplitter (Linos, Germany) and then into wavelength ranges above and below 595 nm by using a dichroic beamsplitter (z595 DCXR, Chroma, USA) before being detected using four avalanche photodiodes (PDM50C, MPD, Italy) for emission wavelengths < 595 nm and SPCM-AQRH-14, Perkin Elmer, USA, for emission wavelengths > 595 nm. For the FCS measurements reported, the use of two APDs along the < 595 nm emission path allowed simultaneous independent measurement of parallel and perpendicular components of the fluorescence (relative to the excitation beam) to be measured. The fluorescence was detected after passing through a bandpass filter (HQ 525/50 nm, Chroma, USA). The signals from both detectors were routed to a digital autocorrelator (ALV-7002, ALV GmbH, Germany) connected to a PC and cross-correlated. 5 million photons were typically collected for each correlation measurement with count rates of ca. 100 kHz. All measurements are reported for a temperature of 21 ± 1 °C. Cross-correlation functions, G(τ), were fitted to equation 1.

$$G(\tau) = C + \left(\frac{1}{N}\right).\left(\frac{1}{1+\frac{\tau}{|\tau_D|}}\right).\left(\frac{1}{\sqrt{1+\frac{\tau}{V^2.|\tau_D|}}}\right).\left(1-|t_f|+t_f.e^{-\frac{\tau}{|t_s|}}\right) \quad \textbf{(Equation 1)}$$

where C is a constant, τ is the lag time, N is the number of molecules in the confocal volume, τ_D is the translational diffusion time, V is a measure of the detection volume defined as z_0/ω_0, where z_0 and ω_0 are the distances at which the 3D Gaussian volume has decayed to $1/e^2$ in the axial and radial directions, respectively, t_f is the triplet fraction and t_s is the triplet lifetime [1].

The translational diffusion time is subsequently related to the diffusion coefficient, D, via equation 2.

$$\tau_D = \frac{\omega_0^{\ 2}}{4D}$$ **(Equation 2)**

The hydrodynamic radius of the diffusing molecule, R_H, may then be found via the Stokes-Einstein relation (equation 3) [2],

$$D = \frac{k_B T}{6\pi\eta R_H}$$ **(Equation 3)**

where k_B is Boltzmann's constant, T is the temperature of the medium and η is the micro-viscosity of the medium.

Fitting parameters associated with the cross-correlation curves of freely-diffusing Rhodamine 110, Alexa 488 (free dye) and labelled disaccharide **6** molecules in 20 mM Tris, 10 mM $MgCl_2$, pH 7.5 buffer are shown in Table S1.

Table S1. Fitting parameters associated with the cross-correlation curves of freely-diffusing Rhodamine 110, Alexa 488 (free dye) and labelled disaccharide **6** molecules in 20 mM Tris, 10 mM $MgCl_2$, pH 7.5 buffer.

	**Rhodamine 100**	**Alexa 488**	**Disaccharide 6**
N	4.37 ± 0.03	6.73 ± 0.06	3.60 ± 0.03
τ_D (μs)	200 ± 2	254 ± 3	307 ± 4
V	3.94 ± 0.04	3.94 ± 0.04	3.93 ± 0.04
t_f	0.06 ± 0.01	0.11 ± 0.01	0.12 ± 0.01
t_s (ms)	0.002	0.003	0.004

Multi-parameter confocal fluorescence spectroscopy

For single-molecule measurements in solution, we used a home-built multiparameter fluorescence detection (MFD) setup, which is based around a confocal microscope with photon-counting detection (Becker and Hickl) and pulsed laser excitation (Picoquant), allowing the simultaneous measurement of fluorescence intensity, color, lifetime and polarization; details of the setup and procedure are found elsewhere [3, 4]. Briefly, the Alexa 488 was excited by a linearly polarized laser (480 nm, 40 MHz, ~ 60 ps FWHM; Picoquant, Germany). The laser light was focused into the dilute solution of labeled molecules by a water immersion objective (UPLAPO 60x, NA=1.2, Olympus, UK). The average laser power of the focused beam at the sample was ca. 170 μW. This photon train is divided into parallel and perpendicular components

via a polarizing beamsplitter (Linos, Germany) and then into wavelength ranges above and below 595 nm by using a dichroic beamsplitter (z595 DCXR, Chroma, USA). Additionally, red (HQ 710/130 nm, AHF Analysentechnik, Germany) and green (HQ 525/50 nm, Chroma, USA) bandpass filters in front of the detectors ensure that only fluorescence photons are registered. Detection is performed using four avalanche photodiodes (SPCM-AQRH-14, Perkin Elmer, USA for the red and PDM50C, MPD, Italy for the green). The signals from all detectors are routed, via delay lines, to a time-correlated single photon-counting board (SPC 132, Becker and Hickl, Germany) connected to a PC.

All measurements were recorded in 20 mM Tris, 10 mM $MgCl_2$, 1mM vitamin C, pH 7.5 buffers at 21 ± 1°C. Measurement buffers were cleaned using activated charcoal prior to use. The lifetime (τ) and steady-state fluorescence anisotropy (r) parameters for MFD were calculated using software written by the group of Prof. Claus Seidel (Heinrich Heine Universität, Düsseldorf as described in detail elsewhere [4]. Sub-ensemble analysis also used software from the Seidel group and involved combining the data from all of the molecules within a specific region of a 2D MFD plot to create a sub-ensemble dataset. In Fig. 1e, the time-resolved decay for the total signal in the green channels (constructed by combining parallel and perpendicular data) for the sub-population indicated was analyzed using DAS6 software from HORIBA Jobin Yvon. Quality of fit or residuals were not improved by fitting to a more complicated decay function. Sample dilutions were prepared in Nunc Lab-Tek II chambered cover glasses, #1,5 (Thermo Fisher, UK).

Encapsulation of Alexa488-labelled Disaccharide in Lipid Vesicles

Small unilamellar vesicles were prepared by the extrusion method [5, 6]. Briefly, a solution of 98 mol% L-α-phosphatidylcholine (Egg, Chicken) (Egg-PC) and 2 mol% 1,2-dipalmitoyl-*sn*-glycero-3-phosphoethanolamine-N-(cap biotinyl) (sodium salt) (Biotinyl-PE) in chloroform was dried using a flow of nitrogen. All lipids were purchased from Avanti Polar Lipids, USA. The dried lipid film was stored under vacuum for 3-4 hours after which it was rehydrated with 20 mM Tris, 6 % (w/v) D-glucose, 0.04 mg/mL catalase, 1 mg/mL glucose oxidase, 2 mM 6-hydroxy-2,5,7,8-tetramethylchroman-2-carboxylic acid (trolox), pH 8 buffer. For single-molecule encapsulation, 100 nM disaccharide **6** was included. For multi-molecule encapsulation > 500 nM disaccharide **6** was added. After mixing of the hydrated lipids (10 mg/mL) unilamellar vesicles were prepared with a miniextruder (Avanti Polar Lipids, USA) using a polycarbonate membrane of 100 nm pore size. After extrusion, vesicle solutions were stored at

4°C in hydration buffer prior to use. The vesicle size distribution was measured by dynamic light scattering (Zetasizer, Malvern Instruments, UK).

TIRF microscopy

Single-molecule TIRF imaging was performed using objective-type total-internal reflection fluorescence microscopy, where the labeled disaccharide constructs were encapsulated within Egg-PC vesicles. After cleaning, slides and coverslips were treated with aminosilane (Fluorochem) and coated in a monolayer of a 100:1 mixture of MeO-poly(ethylene glycol)-NHS and biotin-poly(ethylene glycol)-NHS (MW = 5000; Iris Biotech, Germany). The biotinylated surfaces were then incubated with 0.2 mg/mL neutravidin (Sigma Aldrich, UK) for 10 minutes and subsequently washed carefully with buffer. Biotnylated Egg-PC vesicles were then flushed onto the neutravidin-coated surfaces at a concentration of 0.01 mg/mL and allowed to incubate for 10 minutes at room temperature. Prior to use the samples were diluted in 20 mM Tris, 6 % (w/v) D-glucose (Sigma Aldrich, UK), 0.04 mg/mL catalase (Sigma Aldrich, UK), 1 mg/mL glucose oxidase (Sigma Aldrich, UK) 2 mM 6-hydroxy-2,5,7,8-tetramethylchroman-2-carboxylic acid (trolox, Sigma Aldrich, UK), pH 8 buffer.

All measurements were performed in microfluidic flow cells. Flow cells containing immobilized vesicles were mounted on an inverted microscope (IX71, Olympus). Excitation light (488 nm Stradus (Laser 2000, UK) diode laser) was provided via the evanescent wave of a totally internally reflected beam. For proper angles of incidence, TIR takes place at the interface between the lower coverslip and the sample [7]. The intensity of the evanescent wave decays exponentially upon penetrating the sample, with a decay length of approximately 100-200 nm. Fluorescence emission was collected using a 100x 1.49 numerical aperture oil-immersion objective lens (Olympus) and separated from scattered excitation via a 500 nm dichroic mirror (Chroma, USA). Fluorescence was subsequently collimated (DV2 Multichannel Imaging System, Photometrics) and imaged onto an EMCCD camera (Evolve, Photometrics). All measurements were performed at room temperature (21 ± 1°C). Approximately 200 visible spots within the field of view were observed. The fluorescence intensities of the spots were detected using a cooled EMCCD camera (Evolve, Photometrics, UK) and TIF movies were recorded using ImagePro-Plus 7.0 software with an exposure time of 50 ms. All TIF movies were analyzed by in-house lab routines written in ImagePro-Plus 7.0. The fluorescence vs. time trajectories were viewed and analyzed using MATLAB procedures written in-house. To extract

the true number of stepwise photobleaching events, we employed a 1D edge detection algorithm as previously described [8]. Briefly, the algorithm constructs a series of scaled derivatives from each raw intensity trajectory before identifying local maxima and minima in the scaled derivatives to identify the presence of sharp edges (bleaching events). By tracking the number of sharp edges identified per trace prior to the fluorescence signal reaching the background intensity, the number of significant bleaching events occurring in the data was evaluated.

Supplementary Figures

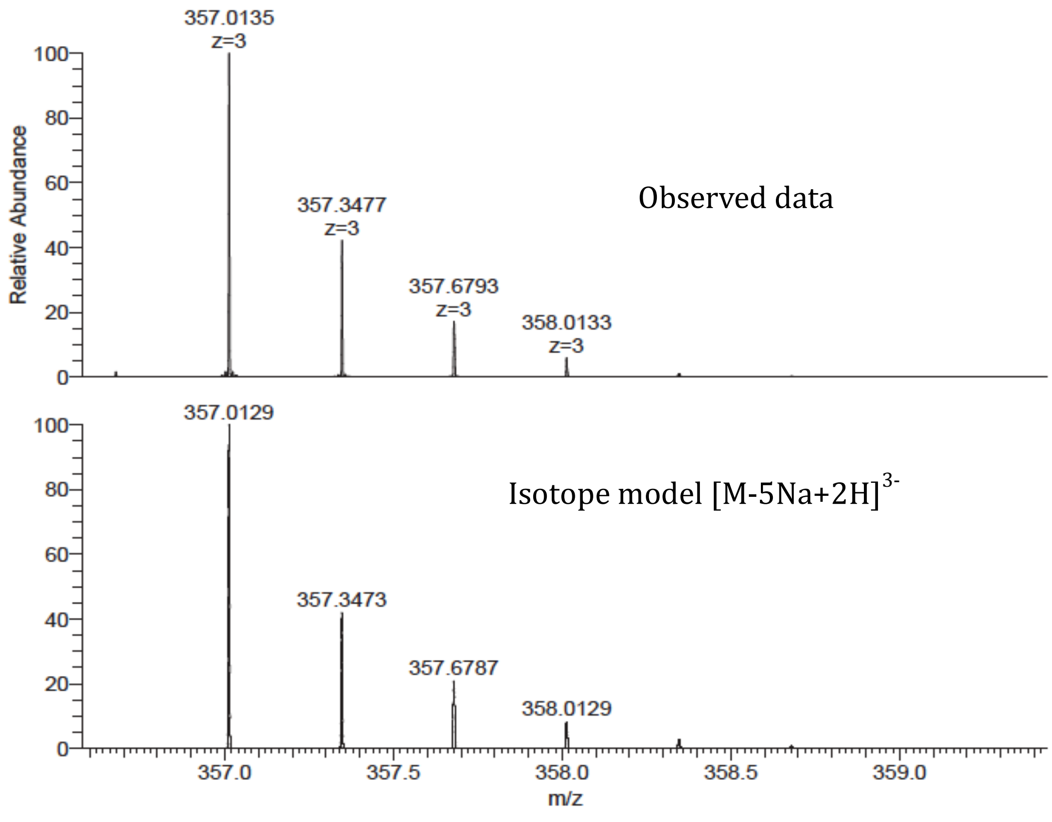

Figure S1. HRMS isotope pattern matching for labelled disaccharide **6** (EPSRC National Mass Spectrometry Facility, Swansea).

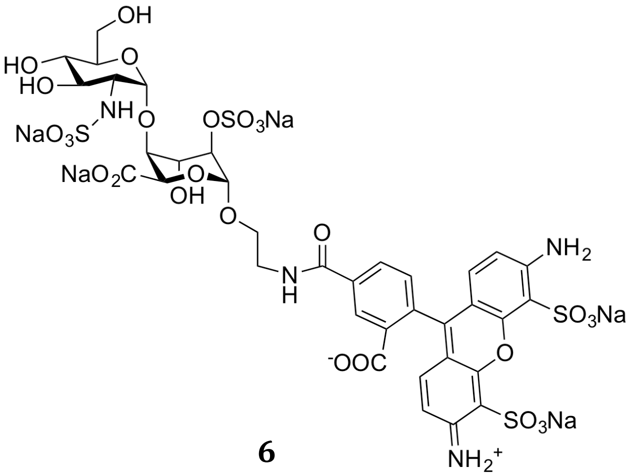

6

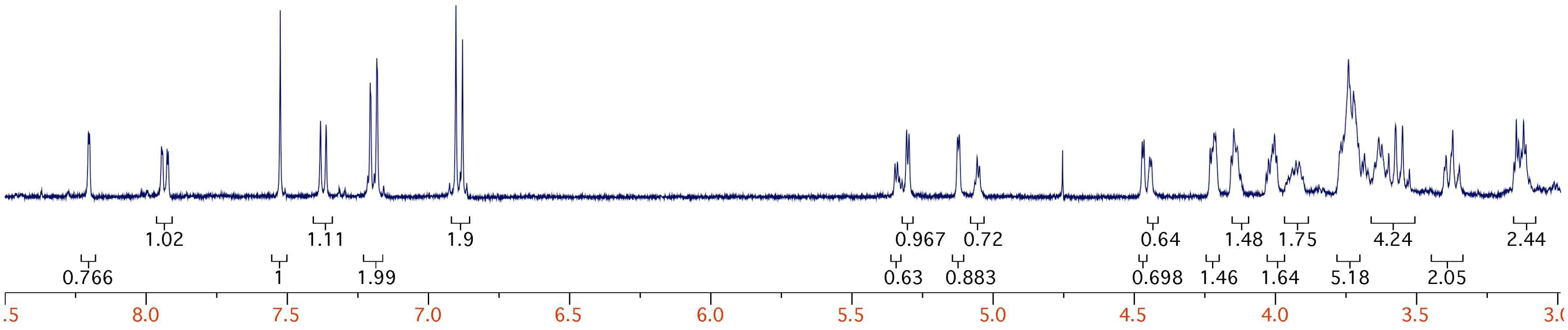

Figure S2. Proton NMR of labelled disaccharide **6** (with unlabelled **5** also present)

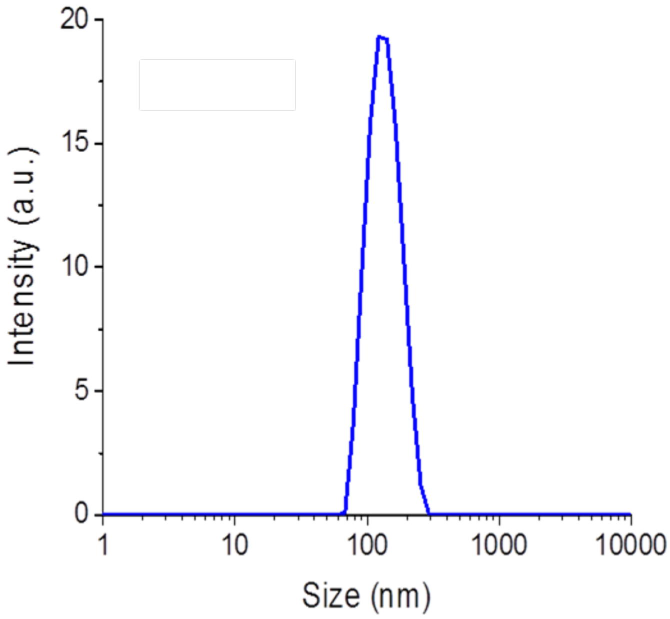

Figure S3. Dynamic light scattering of vesicles. Vesicles were composed of 98 mol% Egg-PC, 2 mol% Biotinyl-PE encapsulating disaccharide **6**.

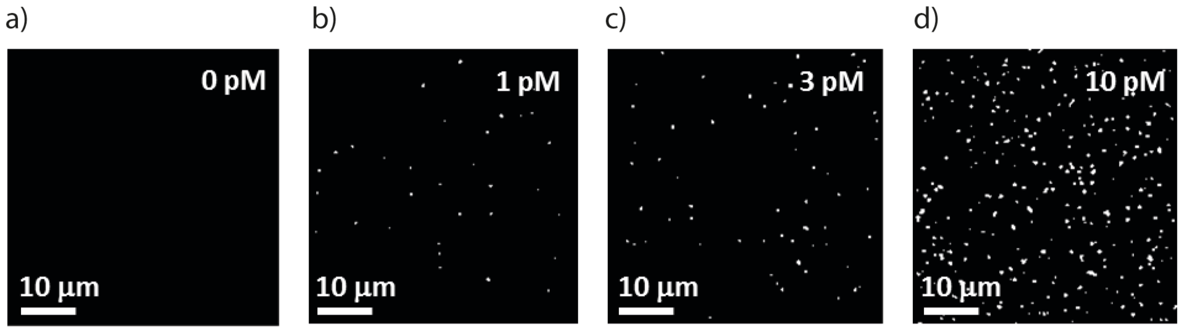

Figure S4. Representative total internal reflection fluorescence (TIRF) images of immobilized vesicles encapsulating disaccharide **6** at concentrations of a) 0 pM, b) 1 pM, c) 3 pM and d) 10 pM.

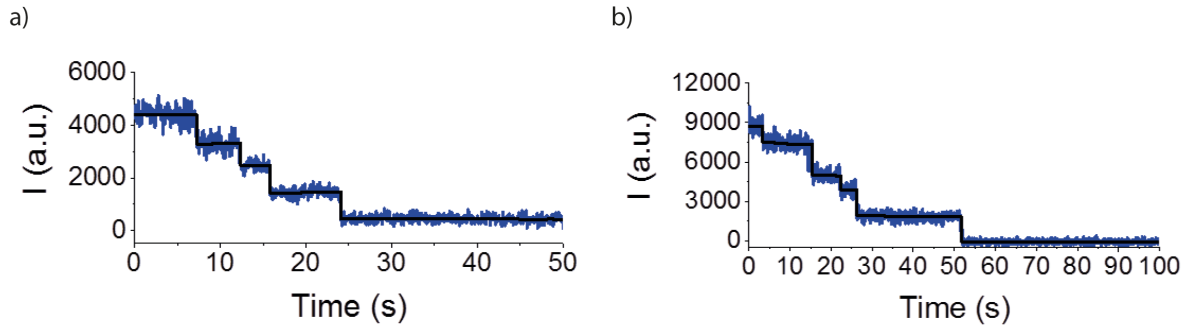

Figure S5. Representative stepwise photobleaching time traces of **6** (1 µM) in individual vesicles encapsulating (a) four and (b) five molecules. Traces were recorded using an integration time of 50 ms and an incident laser power of 65 W/cm^2 at 488 nm. The solid blue lines correspond to the raw data and the solid black lines represent fits acquired using a 1D edge-detection algorithm.

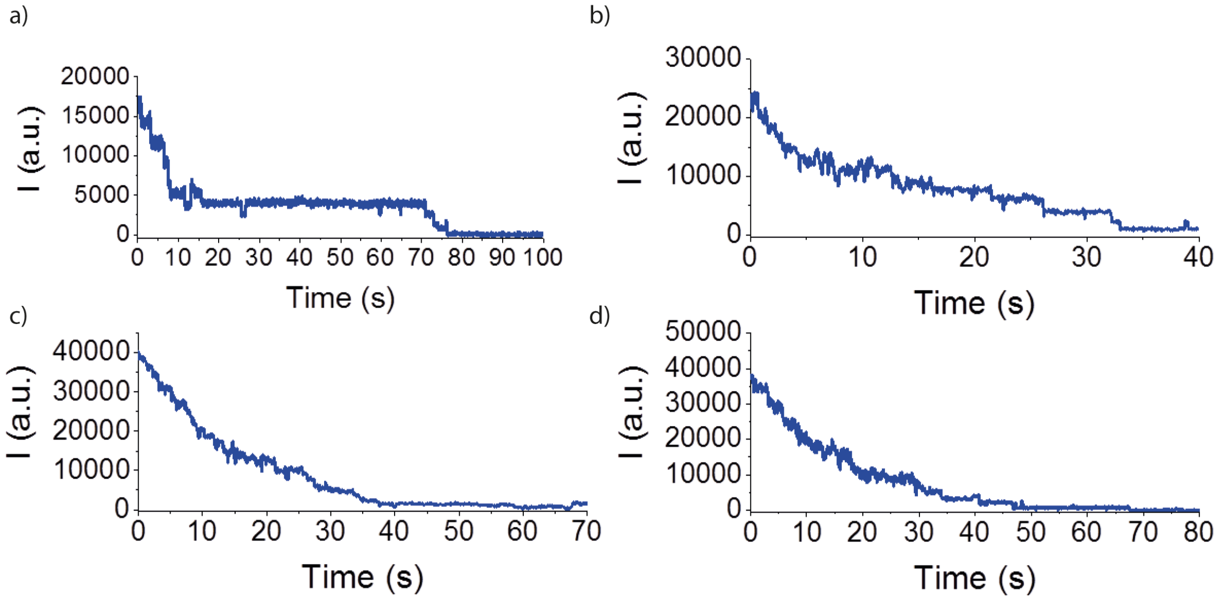

Figure S6. (a)-(d) Representative photobleaching time traces of **6** (10 μM) from individual vesicles encapsulating multiple molecules. Traces were recorded using an integration time of 50 ms and an incident laser power of 65 W/cm^2 at 488 nm.

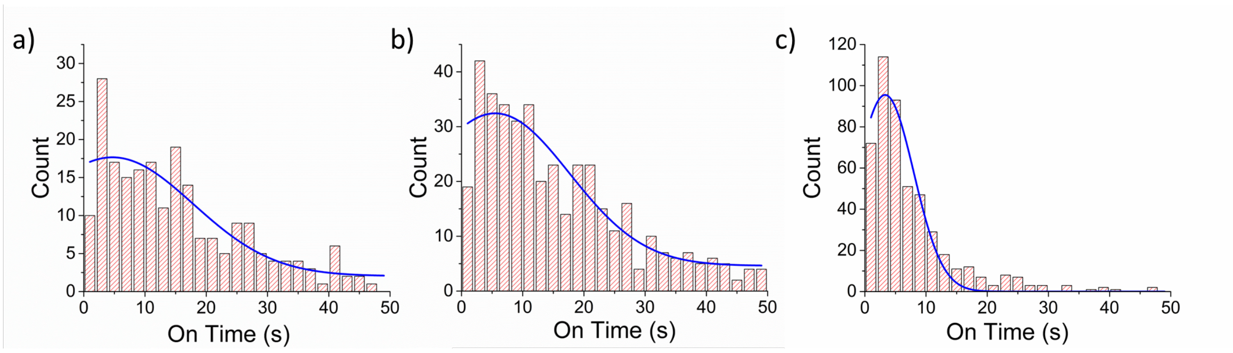

Figure S7. Histograms of the longevities of single disaccharide **6** molecules exposed to laser powers of a) 65 W/cm^2, b) 130 W/cm^2 and c) 260 W/cm^2 at 488 nm. The data for each condition were fitted to Gaussian distributions with center values of 4.6 ± 0.6 s (FWHM = 31 ± 3 s), 5.5 ± 0.5 s (FWHM = 29 ± 3 s) and 3.3 ± 0.5 s (FWHM = 9 ± 1 s), respectively.

NMR Spectra

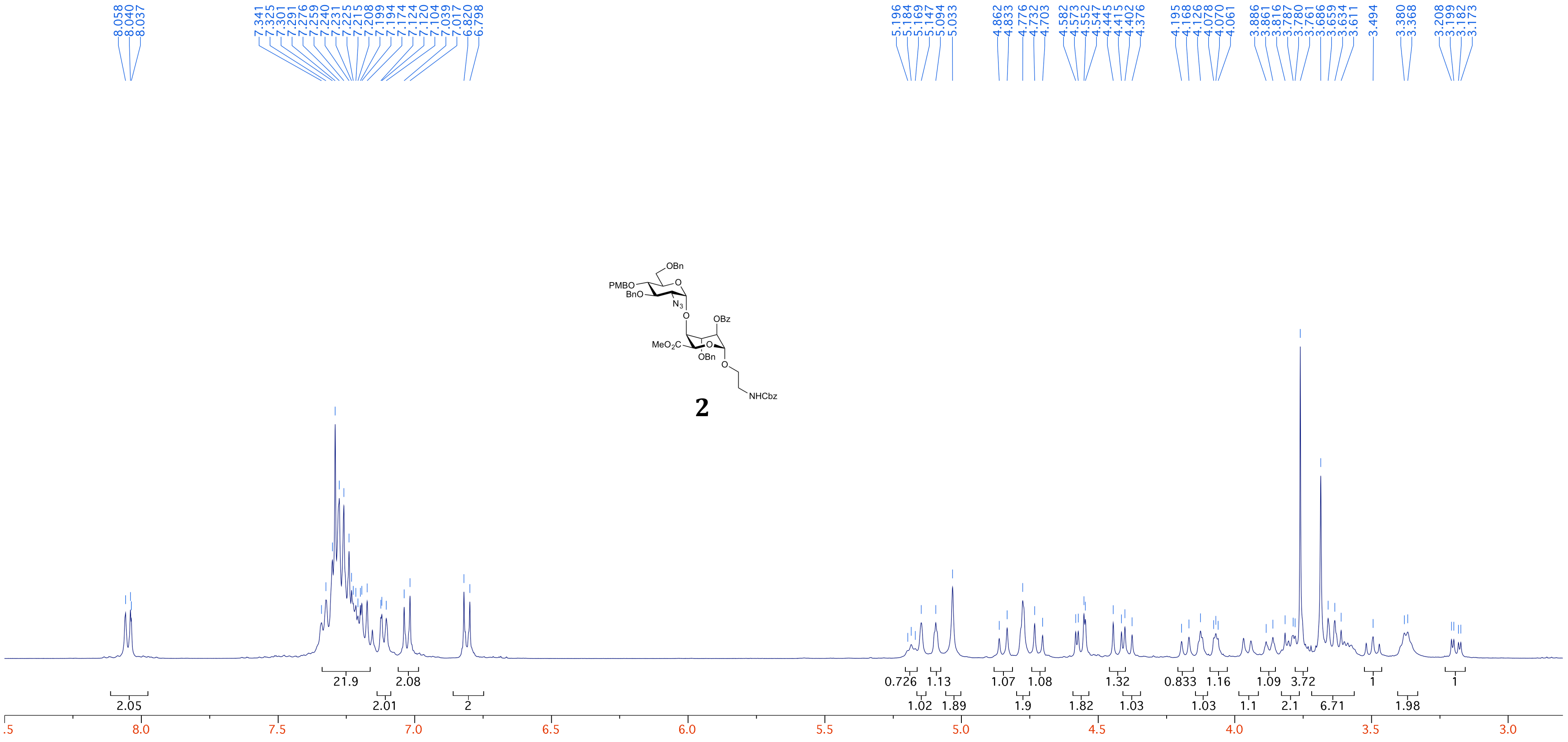

Spectrum 1. Proton NMR of **2**

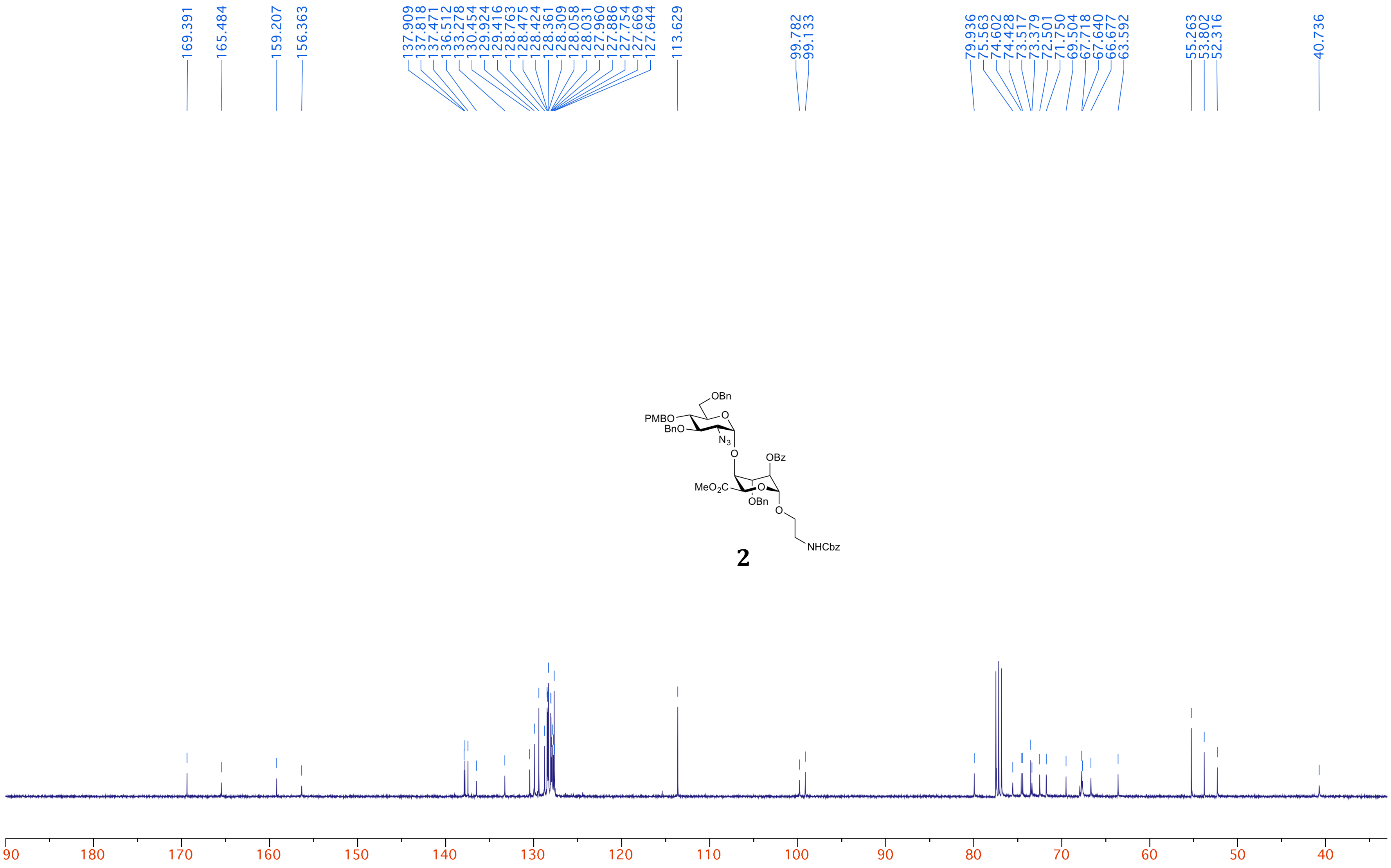

Spectrum 2. ^{13}C NMR of **2**

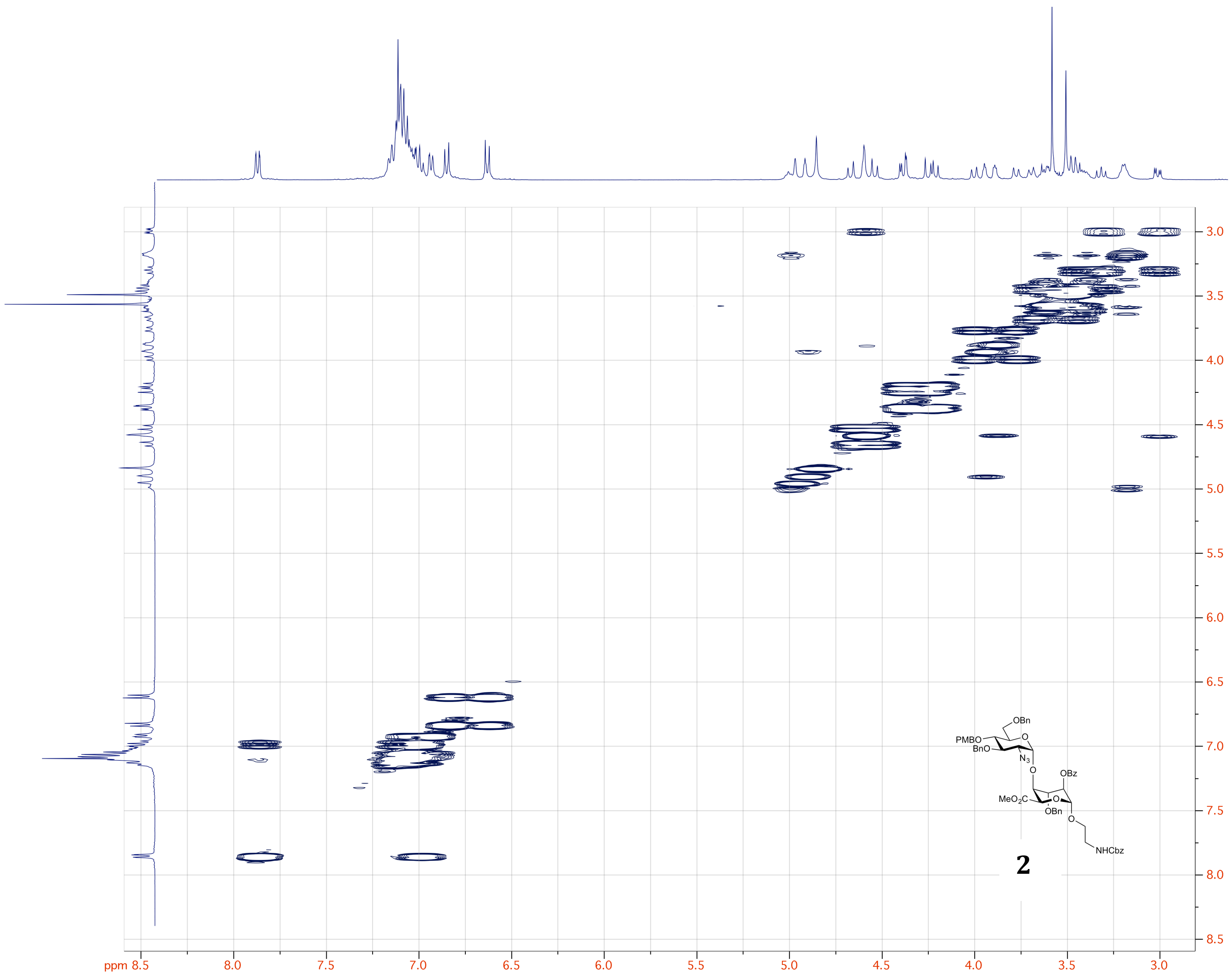

Spectrum 3. COSY of **2**

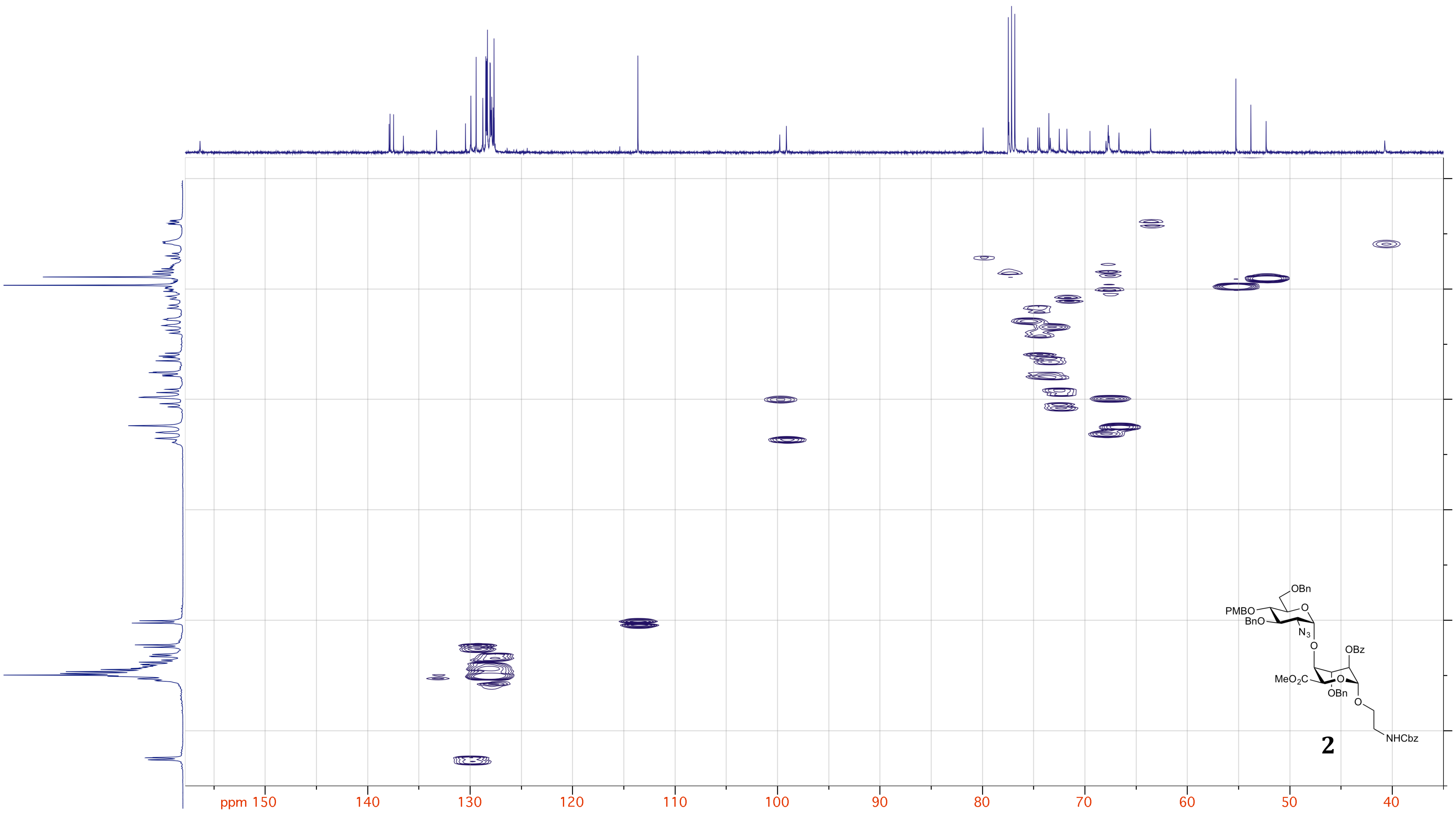

Spectrum 4. HMQC of **2**

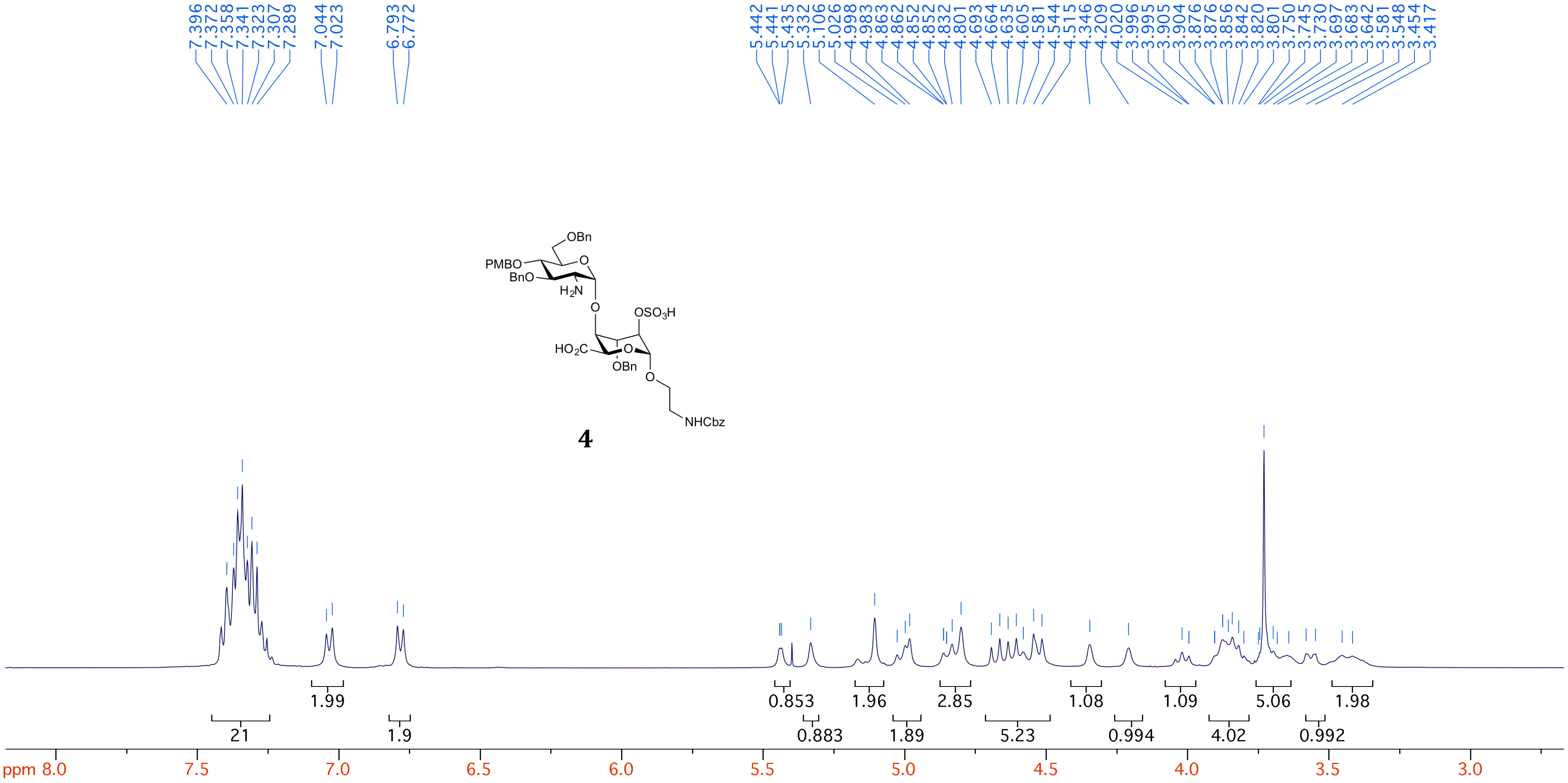

Spectrum 5. Proton NMR of **4**

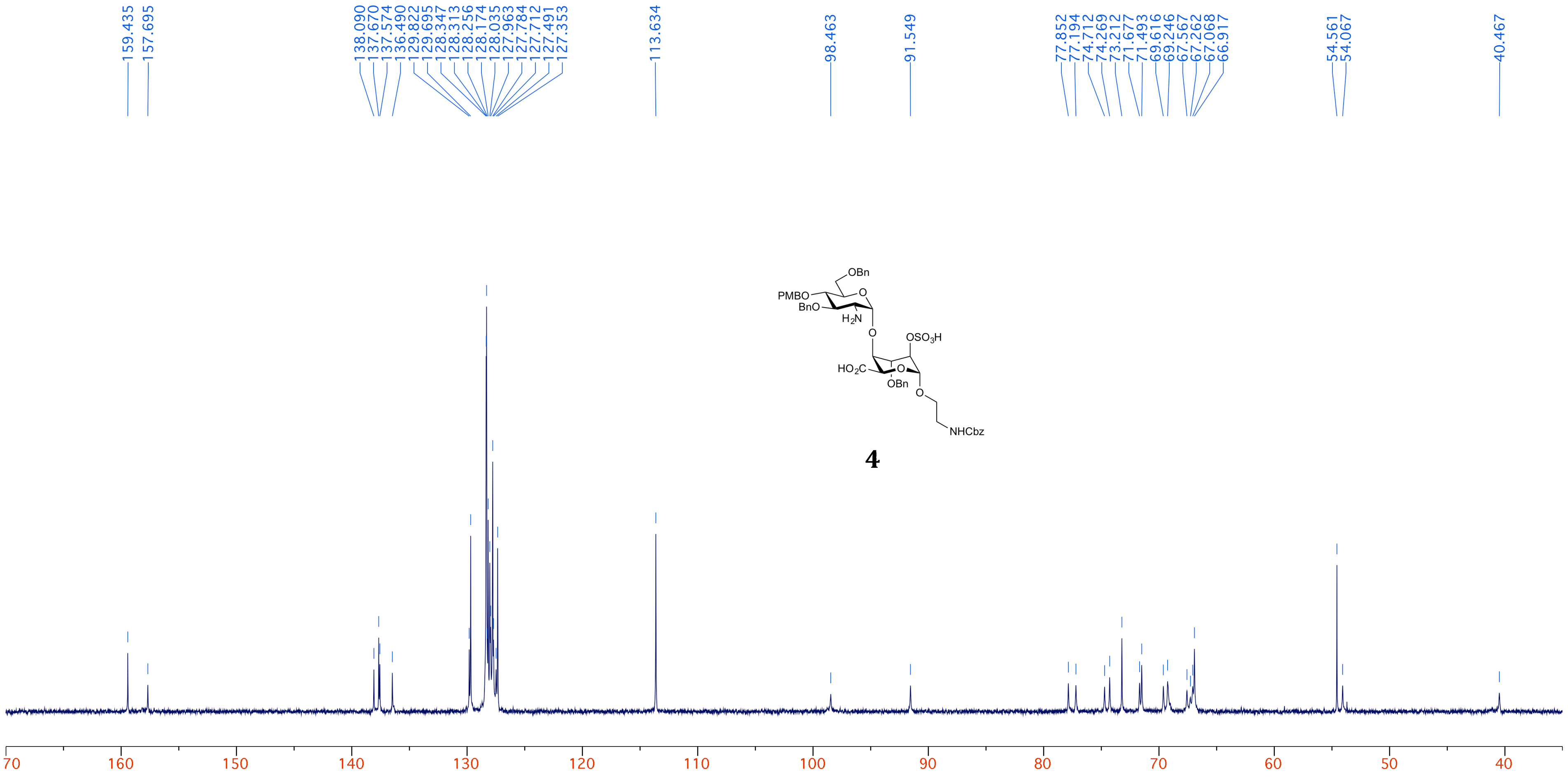

Spectrum 6. ^{13}C NMR of **4**

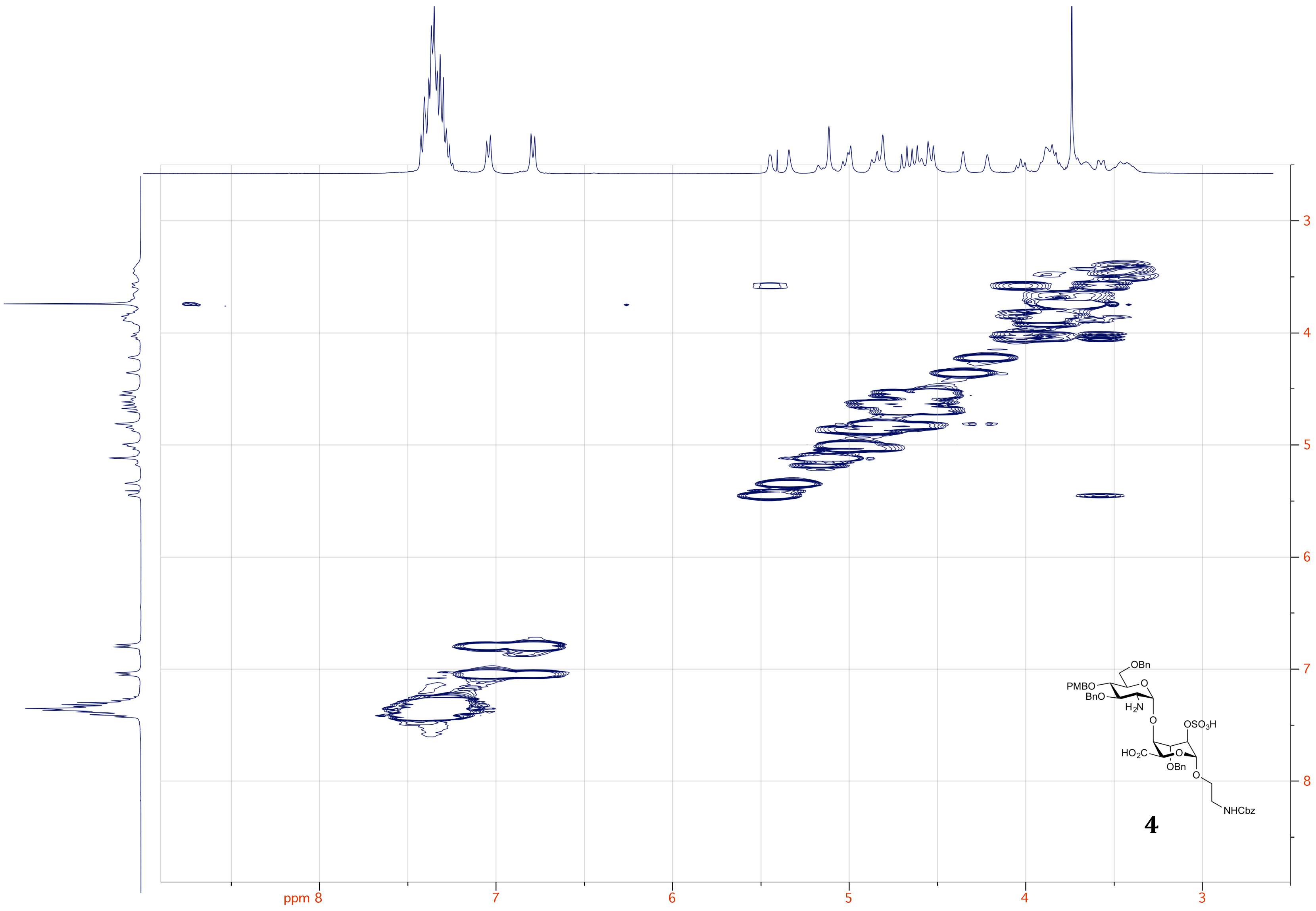

Spectrum 7. COSY of **4**

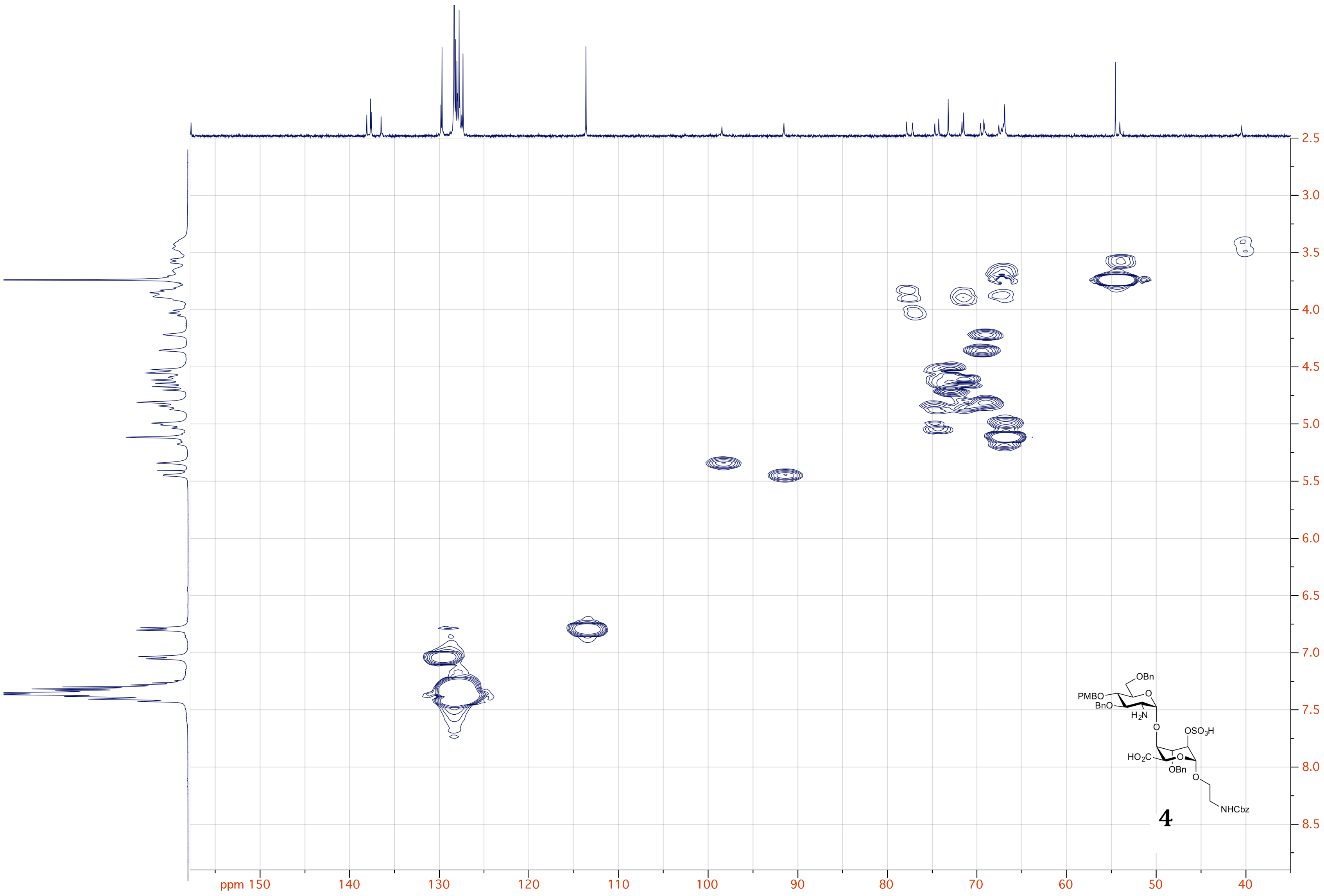

ppm 150
140
130
120
110
100
90
80
70
60
50
40
2.5
3.0
3.5
4.0
4.5
5.0
5.5
6.0
6.5
7.0
7.5
8.0
8.5
OBn
PMBO
BnO
H_2N
O
OSO_3H
HO_2C
OBn
NHCbz
4

Spectrum 8. HMQC of **4**

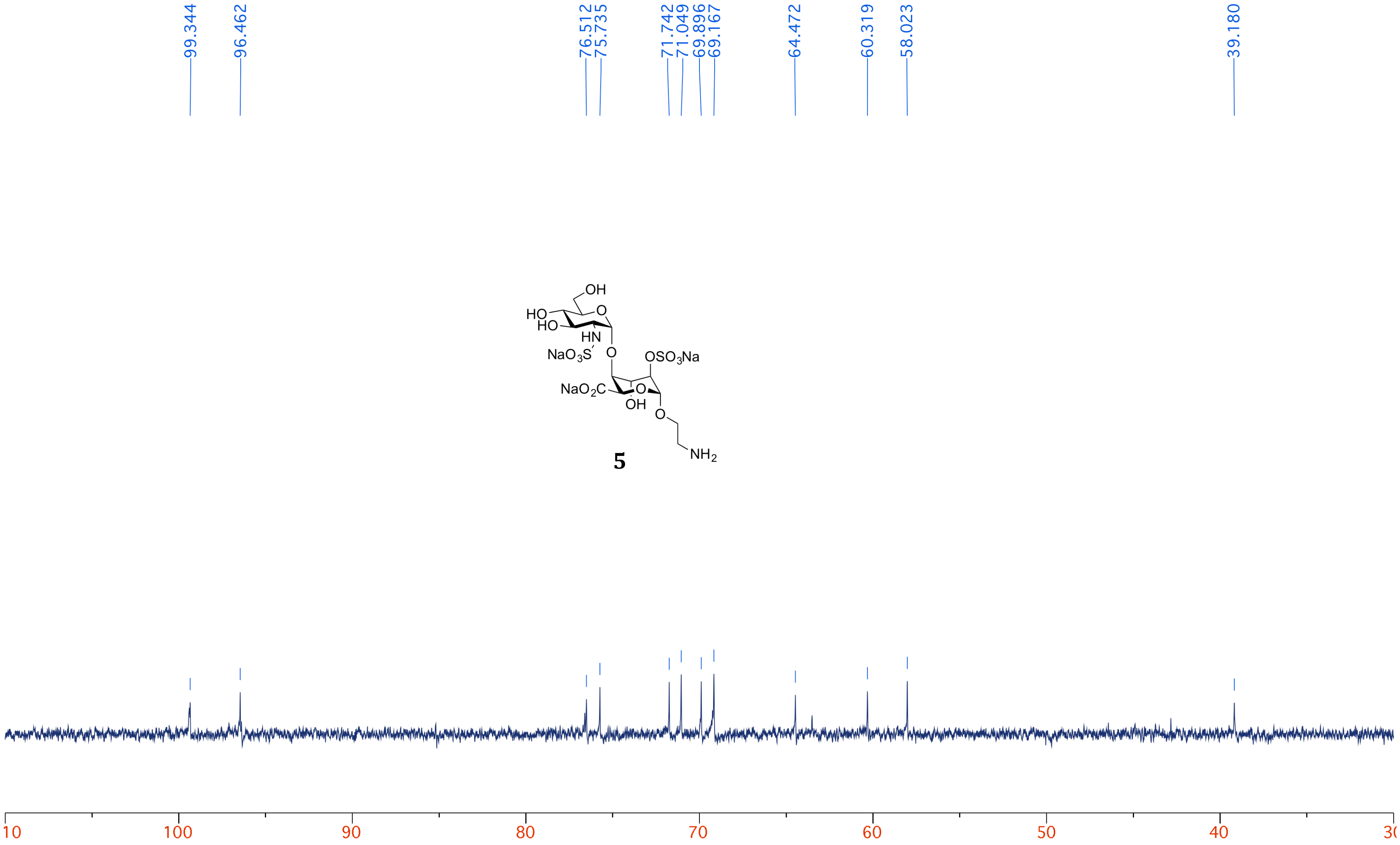

Spectrum 9. ^{13}C NMR of **5**

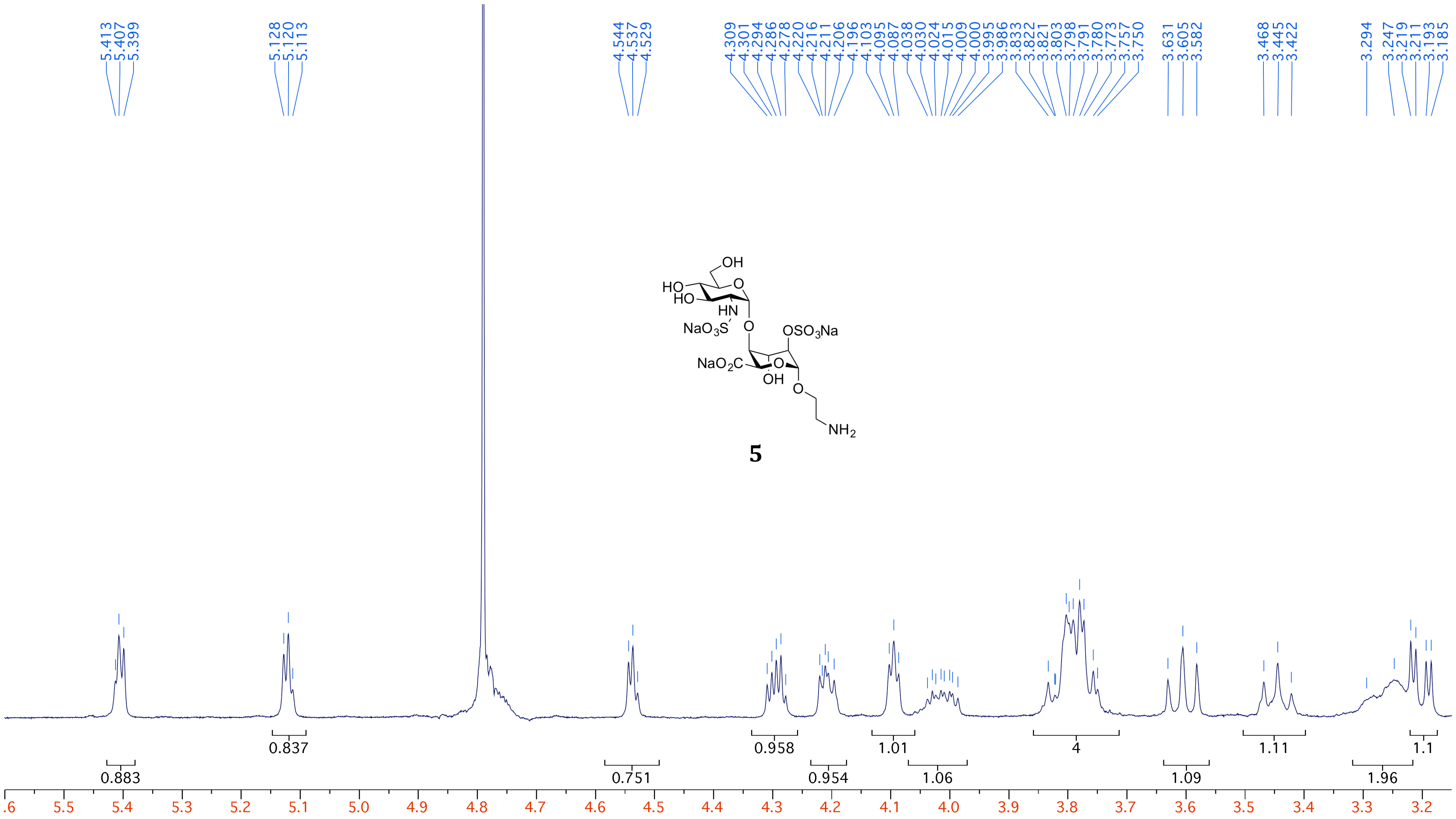

5

Spectrum 10. Proton NMR of **5**

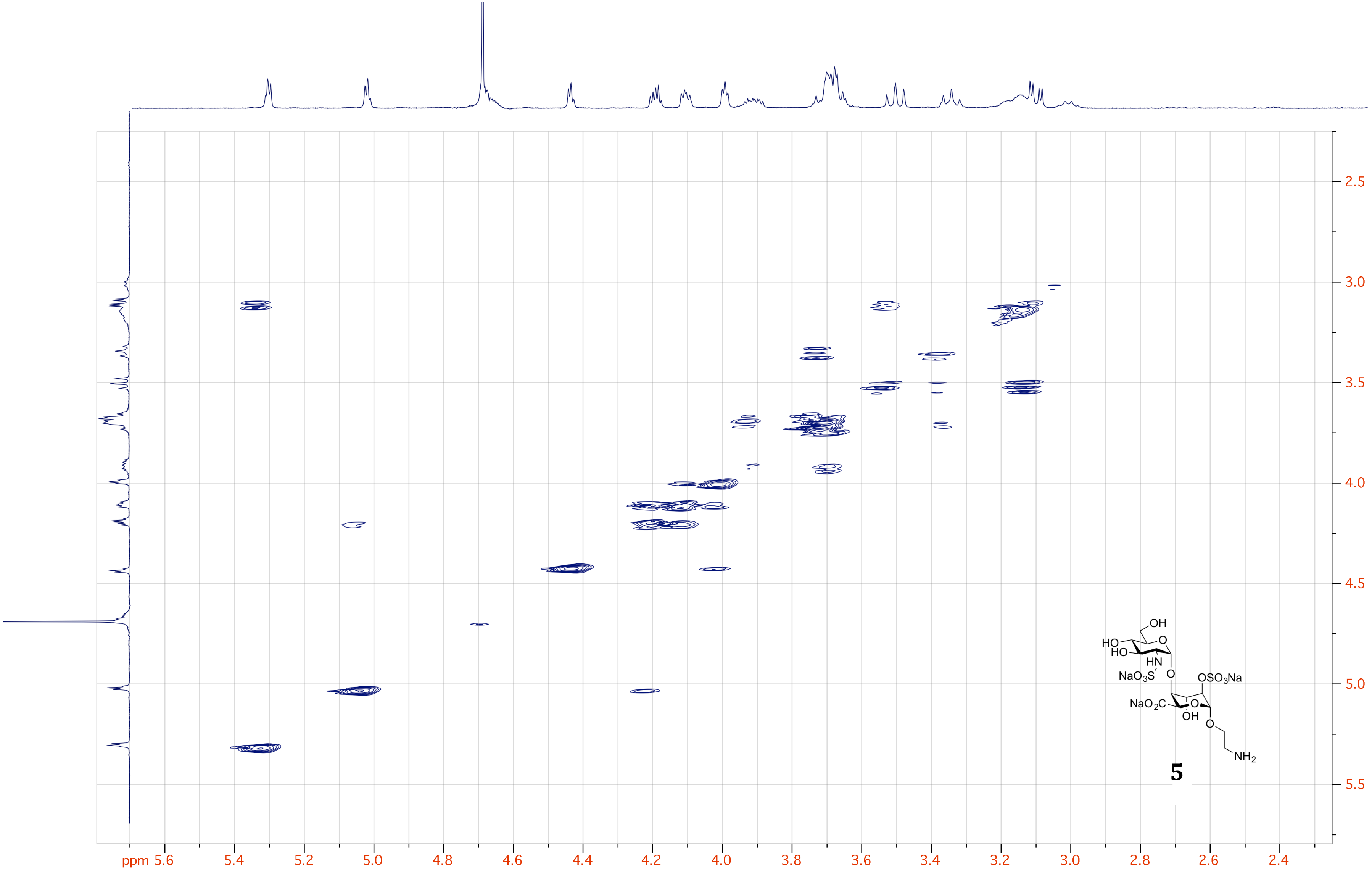

Spectrum 11. COSY of **5**

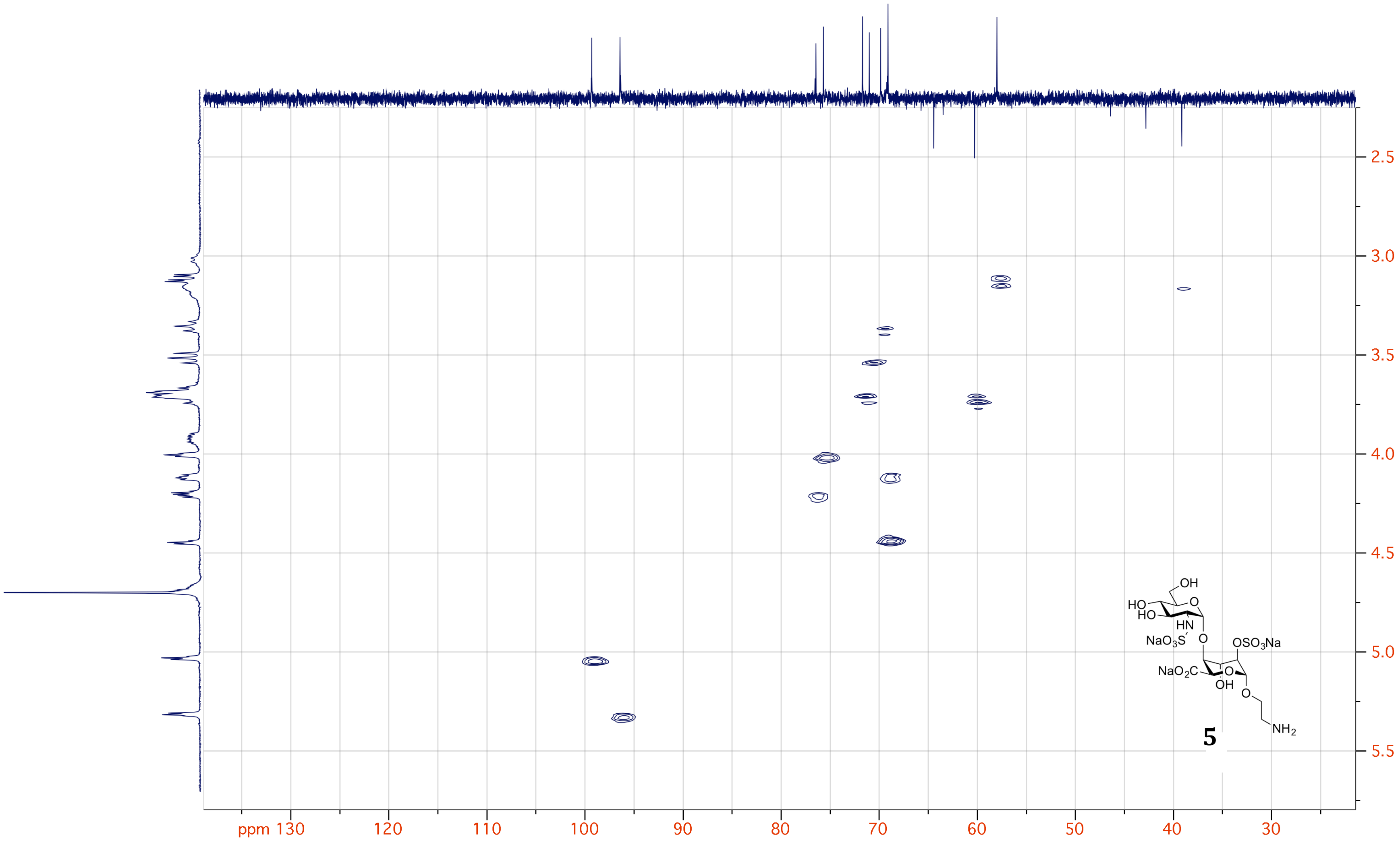
ppm 130
120
110
100
90
80
70
60
50
40
30
2.5
3.0
3.5
4.0
4.5
5.0
5.5
OH
HO
HO
O
HN
NaO3S
O
OSO3Na
NaO2C
O
OH
O
NH2
5

Spectrum 12. HMQC of **5**

References

[1] J. Widengren, U. Mets, R. Rigler. *J. Phys. Chem.* **1995**, 99, 13368-13379.

[2] J. Ries, P. Schwille. *Bioessays.* **2012**, *34*, 361-368.

[3] L. E. Baltierra-Jasso, M. J. Morten, L. Laflör, S. D. Quinn S. W. Magennis. *J. Am. Chem. Soc.* **2015***, 137*, 16020-16023.

[4] E. Sisamakis, A. Valeri, S. Kalinin, P. J. Rothwell, C. A. M. Seidel, *Methods Enzymol.* **2010**, *475*, 455-514.

[5] F. Szoka, D. Papahadjopoulous, *Ann. Rev. Biophys. Bioeng.***1980**, *9*, 467-508.

[6] F. Szoka, D. Papahadjopoulos, *Biochim. Biophys. Acta.* **1980**, *601*, 559-571.

[7] R. Roy, S. Hohng, T. Ha. *Nat. Meth.* **2008**, *5*, 507-516.

[8] J. Canny, *IEEE Trans. Pattern Anal. Mach. Intell.* **1986**, *8*, 679-698.
